# Effective silencing of ENaC by siRNA delivered with epithelial-targeted nanocomplexes in human cystic fibrosis cells and in mouse lung

**DOI:** 10.1136/thoraxjnl-2017-210670

**Published:** 2018-05-10

**Authors:** Aristides D Tagalakis, Mustafa M Munye, Rositsa Ivanova, Hanpeng Chen, Claire M Smith, Ahmad M Aldossary, Luca Z Rosa, Dale Moulding, Josephine L Barnes, Konstantinos N Kafetzis, Stuart A Jones, Deborah L Baines, Guy W J Moss, Christopher O’Callaghan, Robin J McAnulty, Stephen L Hart

**Affiliations:** 1 Experimental and Personalised Medicine Section, UCL Great Ormond Street Institute of Child Health, London, UK; 2 Department of Neuroscience, Physiology and Pharmacology, University College London, London, UK; 3 Institute of Pharmaceutical Science, Faculty of Life Science and Medicine, King’s College London, London, UK; 4 Respiratory, Critical Care and Anaesthesia, UCL Great Ormond Street Institute of Child Health, London, UK; 5 UCL Great Ormond Street Institute of Child Health, London, UK; 6 UCL Respiratory Centre for Inflammation and Tissue Repair, London, UK; 7 Institute of Infection and Immunity, St George’s University of London, London, UK

**Keywords:** airway epithelium, cystic fibrosis, lung physiology, nebuliser therapy

## Abstract

**Introduction:**

Loss of the cystic fibrosis transmembrane conductance regulator in cystic fibrosis (CF) leads to hyperabsorption of sodium and fluid from the airway due to upregulation of the epithelial sodium channel (ENaC). Thickened mucus and depleted airway surface liquid (ASL) then lead to impaired mucociliary clearance. ENaC regulation is thus a promising target for CF therapy. Our aim was to develop siRNA nanocomplexes that mediate effective silencing of airway epithelial ENaC in vitro and in vivo with functional correction of epithelial ion and fluid transport.

**Methods:**

We investigated translocation of nanocomplexes through mucus and their transfection efficiency in primary CF epithelial cells grown at air–liquid interface (ALI). Short interfering RNA (SiRNA)-mediated silencing was examined by quantitative RT-PCR and western analysis of ENaC. Transepithelial potential (V_t_), short circuit current (I_sc_), ASL depth and ciliary beat frequency (CBF) were measured for functional analysis. Inflammation was analysed by histological analysis of normal mouse lung tissue sections.

**Results:**

Nanocomplexes translocated more rapidly than siRNA alone through mucus. Transfections of primary CF epithelial cells with nanocomplexes targeting αENaC siRNA, reduced αENaC and βENaC mRNA by 30%. Transfections reduced V_t_, the amiloride-sensitive I_sc_ and mucus protein concentration while increasing ASL depth and CBF to normal levels. A single dose of siRNA in mouse lung silenced ENaC by approximately 30%, which persisted for at least 7 days. Three doses of siRNA increased silencing to approximately 50%.

**Conclusion:**

Nanoparticle-mediated delivery of ENaCsiRNA to ALI cultures corrected aspects of the mucociliary defect in human CF cells and offers effective delivery and silencing in vivo.

**Key messages**

**What is the key question?**

- Can silencing of the airway epithelial sodium channel (ENaC) activity correct the mucociliary defects associated with the cystic fibrosis (CF) epithelium?

**What is the bottom line?**

- Short interfering RNA (SiRNA)-mediated silencing of ENaC in vitro in pseudostratified, ciliated, air–liquid interface (ALI) models of the human airway was shown to correct the electrical and mucociliary defects associated with the CF epithelium, and in addition, we demonstrated efficiency of delivery in vivo to murine lung and accumulation of the level of silencing by repeated delivery along with safety by analysis of inflammation.

**Why read on?**

- We have described a siRNA nanoparticle formulation that penetrates mucus to transfect CF epithelial cells at ALI, and following repeated transfection both at ALI and in vivo demonstrated cumulative ENaC silencing to achieve the desired functional effect, providing evidence to support its further development as a novel therapeutic approach.

## Introduction

Cystic fibrosis (CF) is caused by mutations in the CF transmembrane conductance regulator gene (CFTR), which encodes a cyclic AMP-activated channel for chloride and other anions.^1 2^ Mutations in CFTR also result in upregulation of the epithelial sodium channel (ENaC), leading to imbalanced water and ion movement across the airway epithelium.^3 4^ This results in depletion of the airway surface liquid (ASL) and thickened mucus with progressive loss of pulmonary function.^3 4^ Therefore, ENaC is a promising therapeutic target for CF with the potential to restore lung fluid homeostasis and thus improve mucociliary clearance. ENaC is comprised of α, β and γ subunits. The pore-forming αENaC subunit is required for full channel function. The β and γ subunits are regulators of ENaC activity,^5 6^ and residual ENaC activity can be measured in their absence.^5 7^ The other ENaC subunit, δ, is only expressed at low levels in the lung of humans and not at all in rodents.^8^

Small molecule ENaC inhibitors such as amiloride^9^ and benzamil^10^ reduce sodium uptake, but their effects are short-lived because of drug absorption.^11^ Silencing of ENaC expression, by short interfering RNA (siRNA)-mediated RNA interference, offers a more promising therapeutic route.^12–14^ Potential advantages of nanoparticle-mediated siRNA therapy include its potency, specificity, duration^15^ and restriction to the airways to prevent nephrotoxicity. Transfection of the CF airway epithelium requires protection of siRNA from nucleases, penetration of the mucus and periciliary liquid layer (PCL) and targeted uptake by the epithelial cells^16^ with purpose-designed nanoparticles. Previous attempts to develop ENaC siRNA therapies^17 18^ were limited by lack of nanoparticles capable to effectively deliver siRNA to the airway epithelium.

Receptor-targeted nanocomplexes (RTNs) comprise multifunctional mixtures of cationic liposomes (L) and cationic targeting peptides (P) that self-assemble, electrostatically on mixing with siRNA (R).^19–24^ Peptide E packages nucleic acids through a cationic, oligolysine domain and mediates epithelial receptor targeting through a seven-amino acid motif, SERSMNF, derived by biopanning of a phage peptide library.^25^ The targeting peptide displays close similarity to receptor binding proteins of two intracellular pathogens, rhinovirus and *Listeria monocytogenes*.^26^ Rhinoviruses bind intercellular adhesion molecule-1 that is present in the airway epithelium and upregulated in the inflamed CF epithelium.^27 28^ Following receptor-mediated endocytosis, the liposome component destabilises the endosomal bilayer allowing nucleic acid release to the cytoplasm before endosomal degradation occurs.^29^ This peptide has been used previously for targeted transfection with plasmid DNA,^25^ minicircle DNA^30^ and siRNA^31^ of bronchial epithelial cells in vitro and in vivo.

In this study, we have investigated the effects of RTN-mediated delivery of αENaC siRNA on pseudostratified, ciliated, air–liquid interface (ALI) models of the human CF airway, particularly the correction of the electrical and mucociliary defects associated with the CF epithelium. Finally, we have investigated the translational potential of this therapy by delivery of murine αENaC siRNA to the lungs of normal mice to assess in vivo transfection efficacy in a surrogate model of the human lung, including efficacy of repeated delivery as well as safety by analysis of inflammation.

## Materials and methods

Full details are available in the online supplementary material.

10.1136/thoraxjnl-2017-210670.supp8 Supplementary data

### Statistics

Data are expressed as the mean±SEM and analysed using a two-tailed, unpaired Student’s t-test or one-way analysis of variance (ANOVA) and Bonferroni’s post hoc analysis where applicable. We also report other data as median and interquartile range (IQR), and these have been analysed using a non-parametric Mann-Whitney U test.

## Results

### Assessment of nanocomplex translocation through mucus

**Figure 1:**
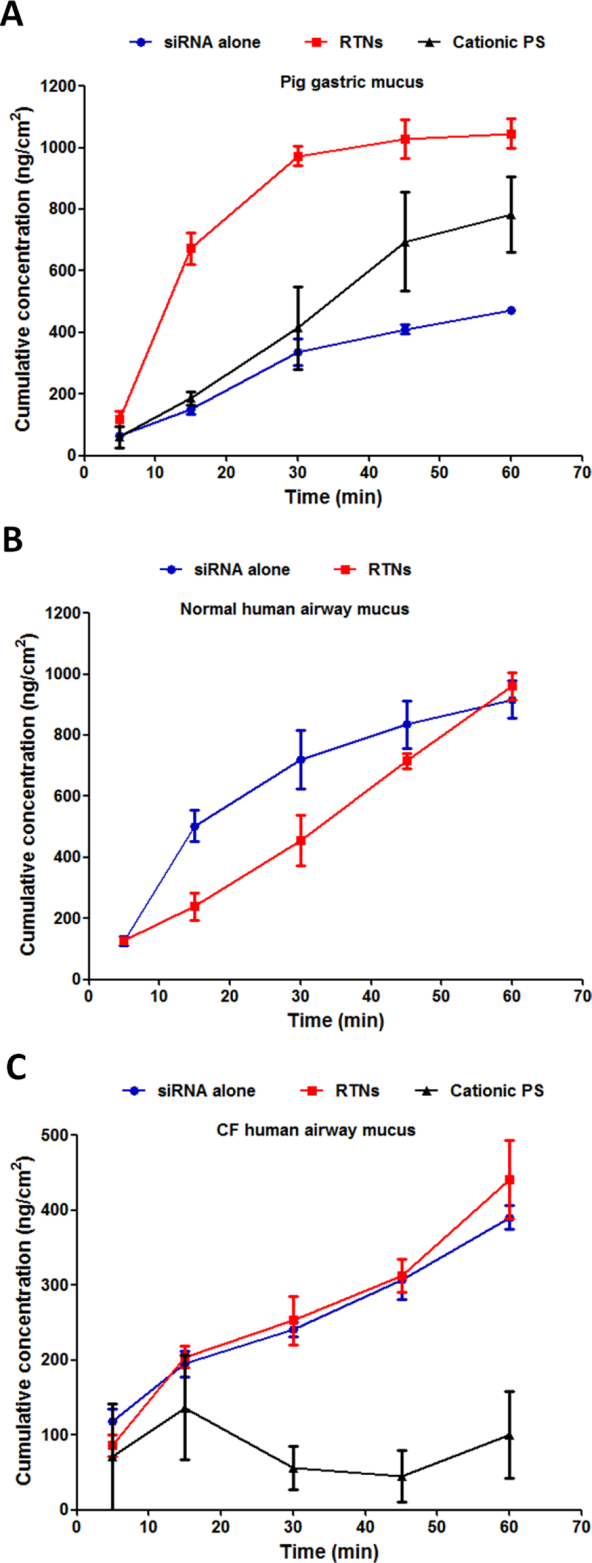
Nanoparticles translocate vertically through the mucus barrier. Cy3-siRNA alone, RTNs containing Cy3-siRNA or fluorescent polystyrene (PS) nanoparticles were investigated for their translocation potential through (A) pig gastric mucus, (B) normal human airway mucus and (C) CF human airway mucus. The siRNA that is labelled with Cy3 is targeting glyceraldehyde 3-phosphate dehydrogenase (GAPDH). The experiments were repeated on three occasions, and each point is the mean±SEM of triplicate measurements. CF, cystic fibrosis; RTNs, receptor-targeted nanocomplexes; siRNA, short interfering RNA.

Nanocomplexes containing Cy3-labelled siRNA were added to the surface of the mucus, then the cumulative concentration of fluorophores diffusing through the mucus into the lower collection chamber over 60 min was measured and compared with siRNA alone and fluorescent cationic, polystyrene (PS) nanoparticles. The concentration of RTNs penetrating through the CF human airway mucus barrier at 1 hour (figure 1C and online supplementary table S1) (441.1±52.7 ng/cm^2^) was 46% of that collected from the normal human airway mucus barrier at 1 hour (961.5±44.8 ng/cm^2^; figure 1B), and this was similar for siRNA alone at 43% (390.1±15.8 ng/cm^2^ through CF human airway mucus and 917.3±60.1 ng/cm^2^ through normal human airway mucus). RTN and siRNA penetration through normal mucus were similar at 1 hour (figure 1B) (p>0.05, n=6), whereas in porcine gastric mucus, the RTNs penetrated more rapidly than siRNA (figure 1A) (1046.1±47.6 ng/cm^2^ and 472.0±8.8 ng/cm^2^ for RTNs and siRNA alone, respectively; p<0.01, n=6). The rate of penetration of pig gastric mucus by cationic PS nanoparticles (55.0±2.1 nm and +23.1±1.1 mV) was not significantly different to siRNA alone (figure 1A; 782.7±123.0 ng/cm^2^ and 472.0±8.8 ng/cm^2^ for PS nanoparticles and siRNA alone, respectively at 1 hour), but in CF mucus, PS nanoparticles were significantly slower than RTNs or siRNA (figure 1C; 100.0±57.6 ng/cm^2^ for PS nanoparticles at 1 hour compared with the concentrations displayed above which were ~4-fold more for both RTNs and siRNA alone; p<0.05, n=6).

10.1136/thoraxjnl-2017-210670.supp7 Supplementary data

Diffusion coefficients in the three types of mucus (D_m_) and water (D_w_) were calculated as described in the Methods section. D_m_/D_w_ is the relative restriction of diffusion in mucus compared with water (table 1). The diffusion of RTNs was 14-fold higher than that of siRNA alone in CF mucus (27-fold impedance for RTNs compared with 376-fold impedance for siRNA alone) despite the nanoparticles being significantly larger (91.9±0.5 nm) and more cationic (+35.1±0.6 mV) than siRNA, which is approximately 7.5 nm.^32^ RTNs penetrated normal human mucus and pig gastric mucus 8.2-fold and 12.3-fold faster, respectively, than siRNA alone (table 1).

**Table 1:** A comparison of the nanoparticle effective diffusion rate in mucus (D_m_) and the effective diffusion rate in water (D_w_) of the receptor-targeted nanocomplexes (RTNs), polystyrene (PS) nanoparticles and siRNA alone through three different types of static layers of mucus. D_m_/D_w_ is the relative restriction of diffusion in mucus compared with water.

Particle	Diffusion in PGM (cm^2^ s^−1^)	D_m_/D_w_ (PGM)	Diffusion in NM (cm^2^ s^−1^)	D_m_/D_w_ (NM)	Diffusion in CFM (cm^2^ s^−1^)	D_m_/D_w_ (CFM)
siRNA	3.75×10^−9^	1.57×10^−2^	1.31×10^−8^	1.11×10^−2^	2.33×10^−9^	2.66×10^−3^
RTNs	1.38×10^−8^	1.93×10^−1^	8.71×10^−9^	9.12×10^−2^	2.67×10^−9^	3.74×10^−2^
PS	5.75×10^−9^	4.82×10^2^	−	−	N/A	N/A

CFM, cystic fibrosis human airway mucus; N/A, data not applicable as effective translocation was not achieved; NM, normal human airway mucus; PGM, pig gastric mucus; PS, polystyrene; RTN, receptor-targeted nanocomplex; siRNA, short interfering RNA.

### In vitro siRNA silencing of ENaC in epithelial cells

Peptide-targeted cationic nanocomplexes were then used to transfect 16HBE14o- epithelial cells with siRNA targeting αENaC. Western blot analysis of αENaC protein showed that transfection of 16HBE14o- cells with RTNs at 75 nM siRNA led to a decrease in the abundance of both the 90 kDa and the 65 kDa αENaC protein bands by 41% and 48%, respectively, compared with those transfected with control siRNA (online supplementary figure S1A,B).

10.1136/thoraxjnl-2017-210670.supp1 Supplementary data

**Figure 2:**
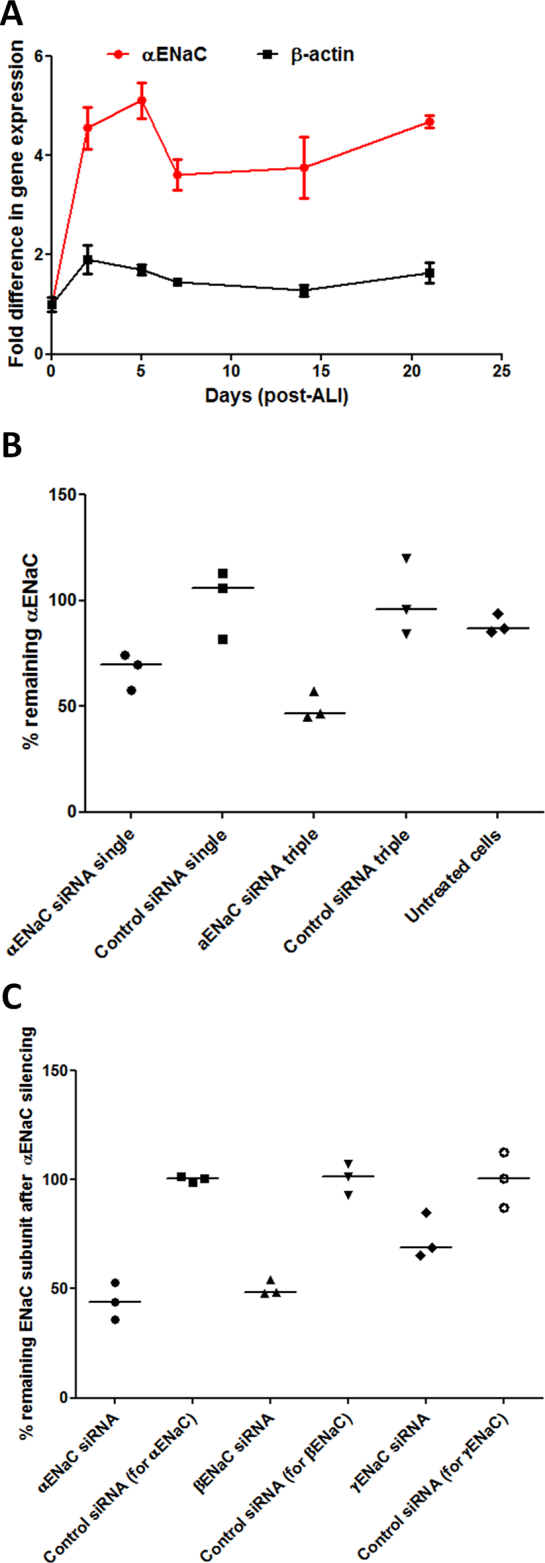
ENaC expression over time of primary cells growing at ALI and silencing of ENaC. (A) The fold-difference in endogenous αENaC gene expression relative to β-actin was quantified by qRT-PCR in CFBE cells from the start date of ALI cultures (mean of n=3 per time point). Points represent mean values±SEM. (B) RTN formulations containing either αENaC siRNA or control siRNA at 100 nM were used in transfections of CFBE cells grown at ALI for 6 weeks. Transfections were performed once (single) or sequentially every other day (triple), and the percentage of silencing was calculated 48 hours after the last transfection (n=3 per formulation). The middle horizontal lines represent the median values. Silencing is normalised to the mean control siRNA set at 100%. (C) CFBE cells grown at ALI were triple-transfected with RTN formulations containing either αENaC siRNA or control siRNA at 100 nM, and the percentage of silencing of α, β and γ ENaC subunits was then calculated 48 hours after transfection (n=3 per formulation). The middle horizontal lines represent the median values. Silencing is normalised to the mean control siRNA set at 100%. Non-parametric Mann-Whitney U tests were performed, and no statistical significant differences were achieved. ALI, air–liquid interface; CFBE, cystic fibrosis bronchial epithelial cells; ENaC, epithelial sodium channel; RTN, receptor-targeted nanocomplex; siRNA, short interfering RNA.

Expression of αENaC in *BMI-1* transduced CF bronchial epithelial (CFBE) cells^33^ attained maximal levels after 2–5 days in ALI cultures (figure 2A), and so, ALI culture transfections in ongoing experiments were performed after at least 5 days ALI culture. Transfections of CFBE monolayers with 100 nM αENaC siRNA reduced αENaC mRNA by 30% (n=3) compared with control siRNA-treated cultures (figure 2B). After three sequential transfections, performed at 48 hours intervals, the level of silencing was improved to 54% (n=3; figure 2B). Silencing of αENaC also resulted in a 51% silencing of βENaC (n=3; online supplementary table S2) but not the γ subunit (figure 2C). The α subunit in CFBE cells was 74.9-fold and 19.7-fold overexpressed relative to the γ and β subunits, respectively (online supplementary figure S2, n=3).

10.1136/thoraxjnl-2017-210670.supp2 Supplementary data

### Functional effects of ENaC silencing

**Figure 3:**
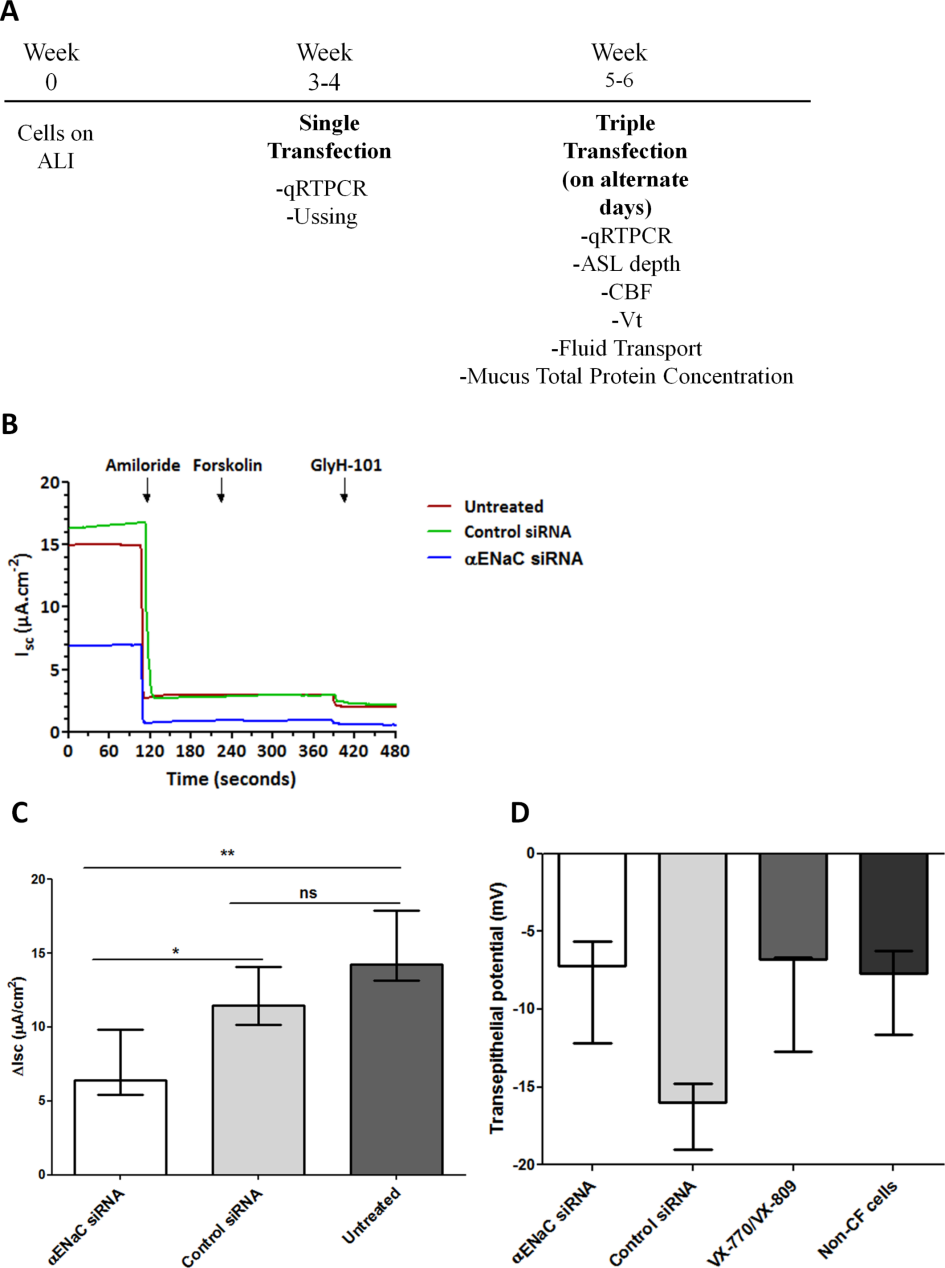
Single transfection of CFBE air–liquid interface (ALI) cell monolayers with nanocomplexes containing αENaC siRNA reduces the amiloride-sensitive short circuit current (I_sc_). (A) A schematic highlighting experiments performed after single or triple transfections on CFBE cells grown at ALI. (B) Representative I_sc_ traces from CFBE monolayers in Ussing chambers of cells treated with 100 nM αENaC siRNA or control siRNA or untreated cells. (C) The change in I_sc_ (ΔI_sc_) after application of 10 µM amiloride is shown for the αENaC siRNA-treated cells (Ussing chambers; n=6), the control siRNA-treated cells (n=8) and the untreated CFBE cells (n=5). Median values are presented as bars and IQR by upper and lower horizontal lines, with statistical significance determined by the non-parametric Mann-Whitney U test. Asterisks indicate comparisons of specific formulations with statistical significance (*p<0.05; **p<0.01). (D) Transepithelial potential (V_t_) of αENaC siRNA-treated monolayers of CFBE cells cultured for 4 weeks on ALI. Wells were triple-transfected with 100 nM αENaC siRNA (n=5) or control siRNA (n=5) and then V_t_ measurements performed 3 days after the last transfection using scanning ion-conductance microscopy (SICM). Further samples were treated with 10 µM VX-809 1 day prior to V_t_ measurement and treated with 10 µM VX-770 during measurement (for approximately 20 min; n=4). Median values are presented as bars and IQR by upper and lower horizontal lines. Non-parametric Mann-Whitney U tests were performed, and no statistical significant differences were achieved. ASL, airway surface liquid; CBF, ciliary beat frequency; CFBE, cystic fibrosis bronchial epithelial cells; ENaC, epithelial sodium channel; siRNA, short interfering RNA.

We next investigated the effects of αENaC silencing on amiloride-sensitive ENaC-mediated short circuit current (I_sc_) in CFBE cells cultured at ALI 2 days after transfection (figure 3A). The amiloride-sensitive I_sc_ was reduced in cells treated with αENaC siRNA (median: 6.4 µA/cm^2^; IQR: 5.4–9.8 µA/cm^2^; n=6) compared with control siRNA (median: 11.5 µA/cm^2^; IQR: 10.1–14.1 µA/cm^2^; p<0.05, n=8) or untreated cells (median: 14.3 µA/cm^2^; IQR: 13.2–17.9 µA/cm^2^; p<0.01, n=5) (figure 3B,C; online supplementary table S3). As expected, CFBE cells showed a very limited response to forskolin (a cAMP agonist) or GlyH-101 (a CFTR inhibitor) under any of the conditions examined (figure 3B) as opposed to normal human bronchial epithelial (NHBE) cells (online supplementary figure S3). Transepithelial electrical resistance (R_t_) was not perturbed by the transfection procedure (online supplementary figure S4), with ENaC-silenced cells having a median of 693.5 (IQR: 565.7–805.8) Ω cm^2^ (n=7) compared with 783.2 (IQR: 711.1–953.2) Ω cm^2^ and 676.7 (IQR: 653.1–744.2) Ω cm^2^ for control siRNA (n=7) and untreated cells (n=3), respectively (online supplementary table S3).

10.1136/thoraxjnl-2017-210670.supp3 Supplementary data

10.1136/thoraxjnl-2017-210670.supp4 Supplementary data

We then performed three sequential siRNA transfections of CFBE cells at 48-hour intervals and determined physiological responses to ENaC silencing. Mucus was not removed during this period (unwashed cells). Samples treated with αENaC siRNA had lower negative transepithelial potentials (V_t_) (median: −7.2 mV; IQR: −5.7 mV to −12.2 mV) than those transfected with control siRNA (median: −16.0 mV; IQR: −14.8 mV to −19.0 mV) (figure 3D; n=5) as measured by scanning ion-conductance microscopy. The median V_t_ value for the VX-770 and VX-809-treated CF cells was −6.8 mV (IQR: −6.7 mV to −12.7 mV), while for untreated, non-CF cells, it was −7.7 mV (IQR: −6.3 mV to −11.6 mV; online supplementary table S4).

**Figure 4:**
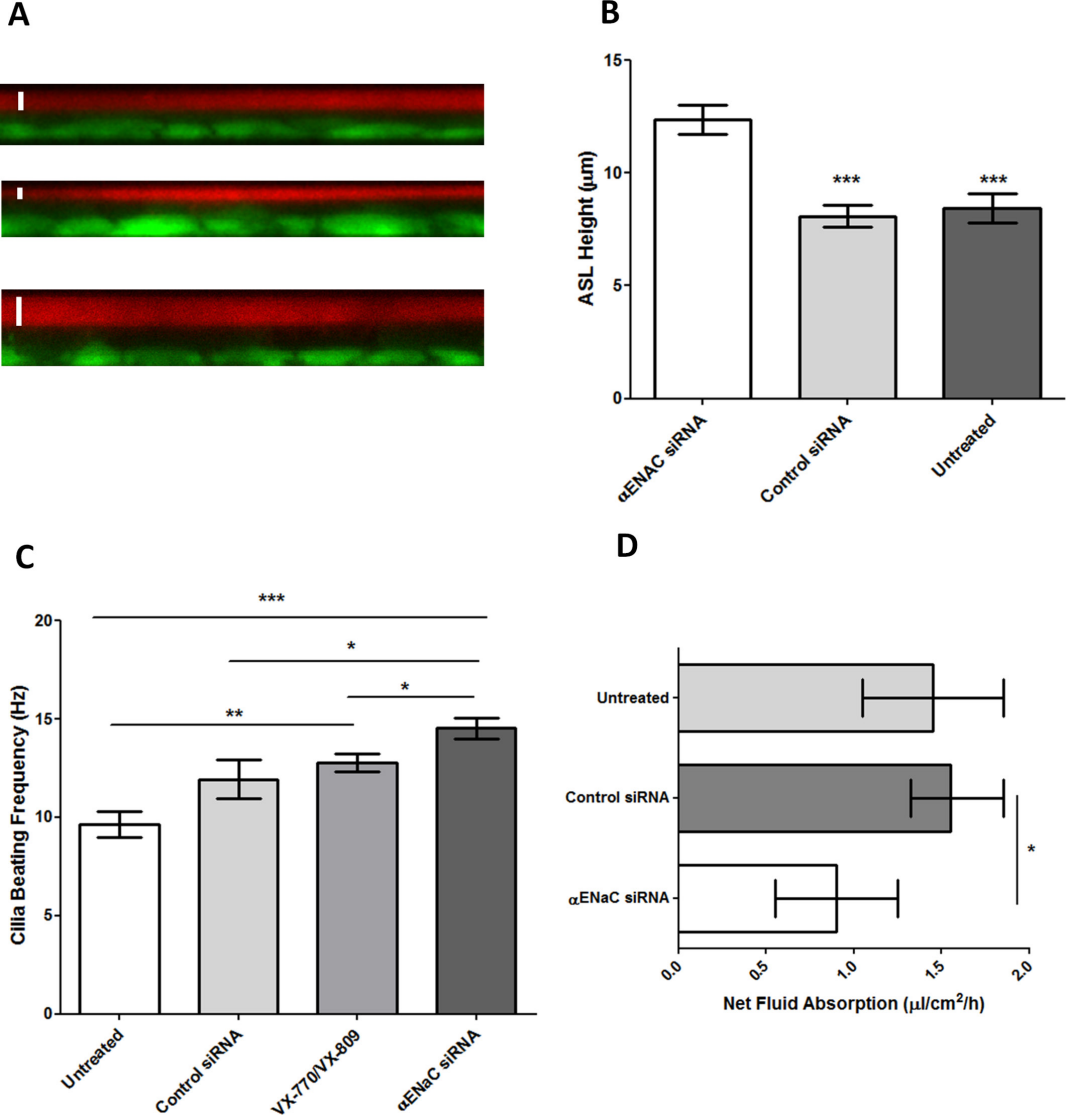
Effect of triple transfections of CFBE ALI cells with nanocomplexes containing αENaC siRNA. The CFBE cells were cultured in snapwells for 4 weeks then treated with 100 nM αENaC siRNA or control siRNA at 48 hours intervals and the measurements performed 3 days after the third dose. (A) ASL depth measurements of CFBE monolayers as determined by confocal microscopy. XZ representative images of fluorescently labelled ASL (red) and cells (green). Top: untreated cells, middle: cells transfected three times with 100 nM of control siRNA and bottom: cells transfected three times with 100 nM of αENaC siRNA. A white bar has been included to denote the ASL measurement. (B) ASL depth measurement of each treatment group (n=4). Bars represent mean±SEM, while asterisks indicate statistical significance of comparisons between the αENaC siRNA-treated cells versus the controls (***p<0.001) determined by an ANOVA test followed by Bonferroni’s post hoc test. (C) Effect of αENaC siRNA, control siRNA and VX-770 and VX-809 on ciliary beat frequency (CBF) of CFBE cells grown at ALI. For each experimental condition (n=10), readings of CBF were calculated from 10 ciliated areas in the snapwell, and the data represent the mean±SEM. Asterisks indicate comparisons of specific formulations with statistical significance (*p<0.05; **p<0.01; ***p<0.001) determined by an ANOVA test followed by Bonferroni’s post hoc test. (D) Transepithelial fluid transport through human CFBE cells. Net fluid absorption rate was measured 4 days after cell treatment with a control siRNA or with αENaC siRNA. Bars represent the median value with IQR shown by the horizontal lines (n=4), while asterisks indicate statistical significance (*p<0.05) determined by non-parametric Mann-Whitney U tests. ALI, air–liquid interface; ASL, airway surface liquid; CFBE, cystic fibrosis bronchial epithelial cells; ENaC, epithelial sodium channel; siRNA, short interfering RNA.

The ASL depth in CFBE ALI cultures was increased by ~1.5 fold (median: 12.1 µm; IQR: 10.7–14.9 µm) after silencing of αENaC compared with control siRNA-treated (median: 7.9 µm; IQR: 6.4–9.8 µm) and untreated cells (median: 8.2 µm; IQR: 5.8–11.1 µm) (p<0.001, n=4) (figure 4A,B; online supplementary table S4). The ciliary beat frequency (CBF) of unwashed CFBE cells increased to 14.5±0.5 Hz from 9.6±0.7 Hz in untreated CFBE cultures following sequential treatment with αENaC siRNA (p<0.001, n=10), compared with 11.9±1.0 Hz in control siRNA-treated cells (p<0.05, n=10) (figure 4C). The CBF of VX-770 and VX-809-treated cells was also increased to 12.8±0.5 Hz (p<0.05, n=10; online supplementary table S4).

**Figure 5:**
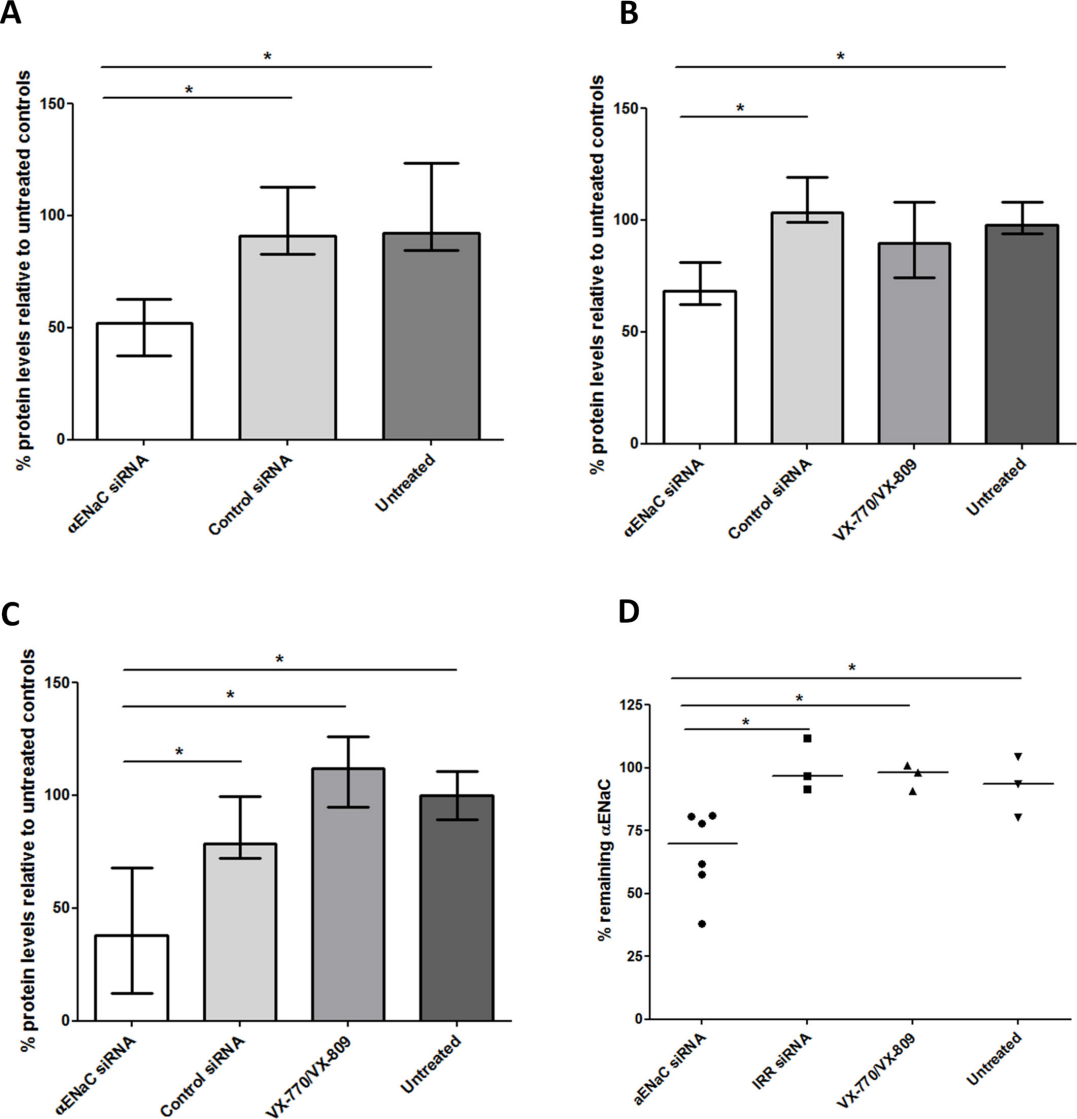
Protein concentration in mucus collected at different time points from transfected or untreated CFBE cells grown at ALI and in vitro silencing after three transfections. Mucus collections were done (A) 2 days after the first transfection (αENaC siRNA treated, n=6; control siRNA treated, n=3; untreated controls, n=3), (B) 2 days after the second transfection (αENaC siRNA treated, n=6; control siRNA treated, n=3; VX-770 and VX-809 treated, n=3; untreated controls, n=3) and (C) 7 days after the third transfection (αENaC siRNA treated, n=6; control siRNA treated, n=3; VX-770 and VX-809 treated, n=3; untreated controls, n=3). The concentration levels were normalised to those of the untreated cells. (D) Formulations containing either 100 nM αENaC siRNA (n=6) or control siRNA (n=3) were used in three sequential transfections of CFBE-BMI-1 cells grown at ALI for 4 weeks, and the percentage of silencing was calculated 8 days after the third transfection. Silencing was normalised to the mean control siRNA set at 100%. The middle horizontal lines represent the median values. Asterisks indicate comparisons of specific formulations with statistical significance (*p<0.05) determined by non-parametric Mann-Whitney U tests. ALI, air–liquid interface; CFBE, cystic fibrosis bronchial epithelial cells; ENaC, epithelial sodium channel; siRNA, short interfering RNA.

Cells treated with αENaC siRNA displayed reduced net fluid absorption rates from the apical side of the epithelium with a median value of 0.9 µL/cm^2^/hour (IQR: 0.6–1.3 µL/cm^2^/hour), which was lower than that of both control siRNA-treated cells (median: 1.6; IQR: 1.3–1.9 µL/cm^2^/hour; p<0.05, n=4) and untreated cells at 1.5 µL/cm^2^/hour (IQR: 1.1–1.9 µL/cm^2^/hour; figure 4D; online supplementary table S4). Finally, we measured the total protein concentration in mucus collected from each well of the sequentially transfected CF monolayers. The apical surface of each well was washed with PBS 2 days after the first (figure 5A) and second transfections (figure 5B) and 7 days after the third (figure 5C) transfection. In all cases, the protein concentration in mucus from the αENaC-silenced cells (n=6) was significantly lower than that from the control siRNA-treated cells (n=6), VX-770 and VX-809-treated cells (n=4) or untreated cells (n=3), and this effect lasted for at least 7 days post-transfection. At the end of the experiment, at 8 days post-transfection with αENaC siRNA (n=6), αENaC expression was still 27% lower than control siRNA-treated cells (p<0.05; figure 5D).

### In vivo lung delivery

**Figure 6:**
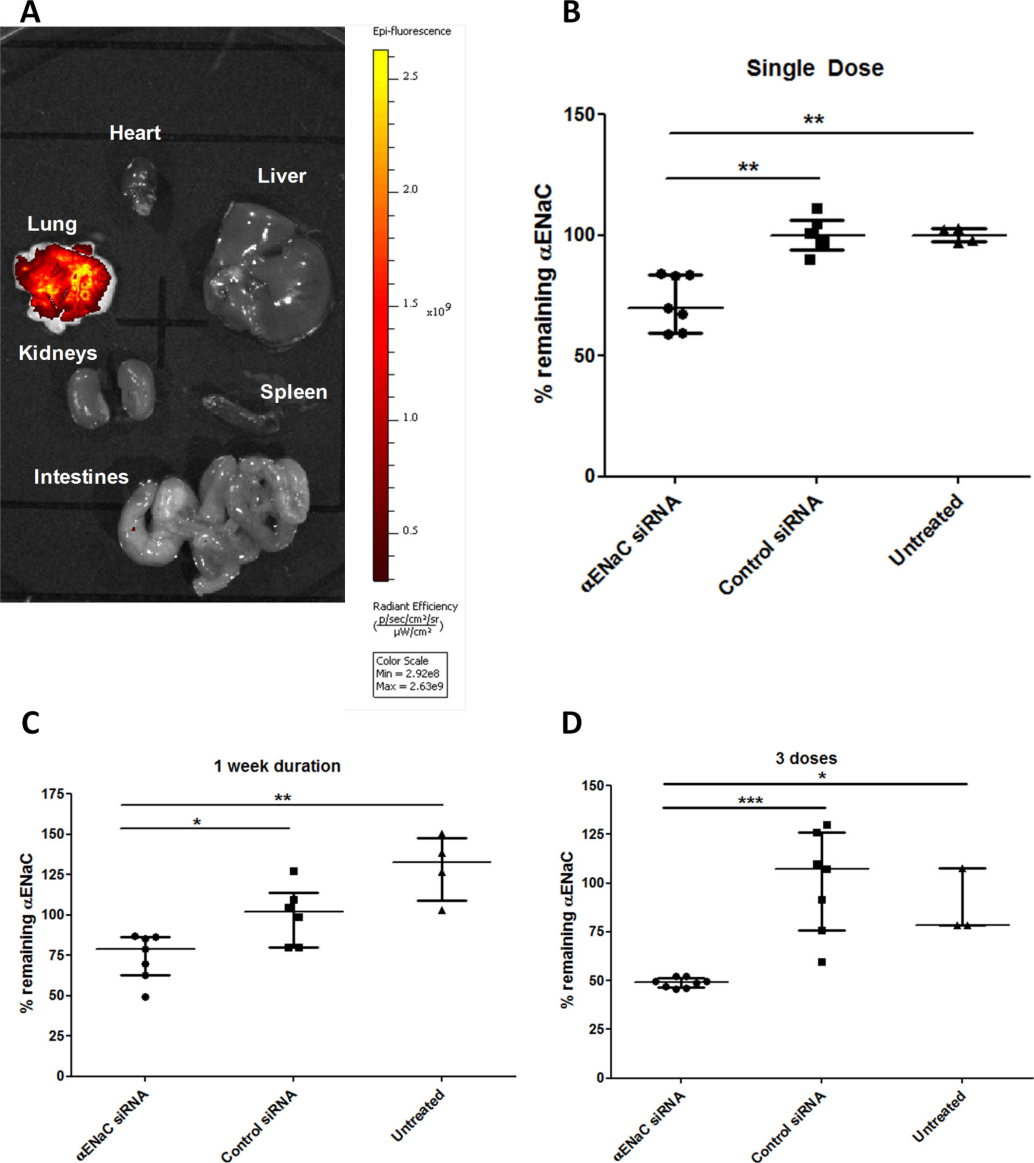
In vivo lung administration of siRNA-containing nanocomplexes. (A) Uptake of Dy677-siRNA formulations following oropharyngeal administration. Twenty-four hours later, the mice were culled (n=3 per group), and organs (heart, lung, liver, kidneys, spleen and intestines) were extracted and imaged for fluorescence. (B–D) The remaining αENaC mRNA was quantified by qRT-PCR in C57BL6 female mice after instillation of cationic nanocomplexes containing 16 µg αENaC siRNA (n=7) or control siRNA (n=6) at (B) 48 hours and (C) 7 days. (D) The amount of remaining αENaC mRNA detected by qRT-PCR in C57BL6 female mice at 72 hours after the last of three instillations of cationic nanocomplexes containing 16 µg αENaC siRNA (n=8) or control siRNA (n=7). Silencing was normalised to the mean control siRNA set at 100%. Medians and IQRs are presented by horizontal lines. Asterisks indicate comparisons of specific formulations with statistical significance (*p<0.05; **p<0.01; ***p<0.001) determined by non-parametric Mann-Whitney U tests. ENaC, epithelial sodium channel; siRNA, short interfering RNA.

We then investigated delivery of siRNA into the lungs of normal mice to assess the translational potential of αENaC siRNA delivery. The biodistribution analysis of nanocomplexes containing Dy677-labelled siRNA 24 hours after oropharyngeal instillation showed very high retention of siRNA in lungs (figure 6A) with low-level fluorescence in intestines (online supplementary figure S5, p<0.05, n=3) which probably reflects incidental swallowing during administration. There was no fluorescence in heart, liver, kidneys and spleen suggesting no detectable transfer of the siRNA from the lung to the circulation (figure 6A). Transfecting mice by oropharyngeal instillation with αENaC siRNA (n=7) or control siRNA (n=7) silenced αENaC by 30% at the mRNA level compared with control siRNA (p<0.01) (figure 6B). Silencing of αENaC 1 week after transfection remained at 23% compared with control siRNA (figure 6C; n=4 for untreated, n=6 for control siRNA and n=7 for αENaC siRNA-treated mice) and was not significantly different from silencing at 48 hours. Repeated transfections with αENaC siRNA (n=8) demonstrated cumulative silencing, increasing to 58% reduction compared with control siRNA at 72 hours after the third instillation of αENaC siRNA (figure 6D; online supplementary table S5).

10.1136/thoraxjnl-2017-210670.supp5 Supplementary data

**Figure 7:**
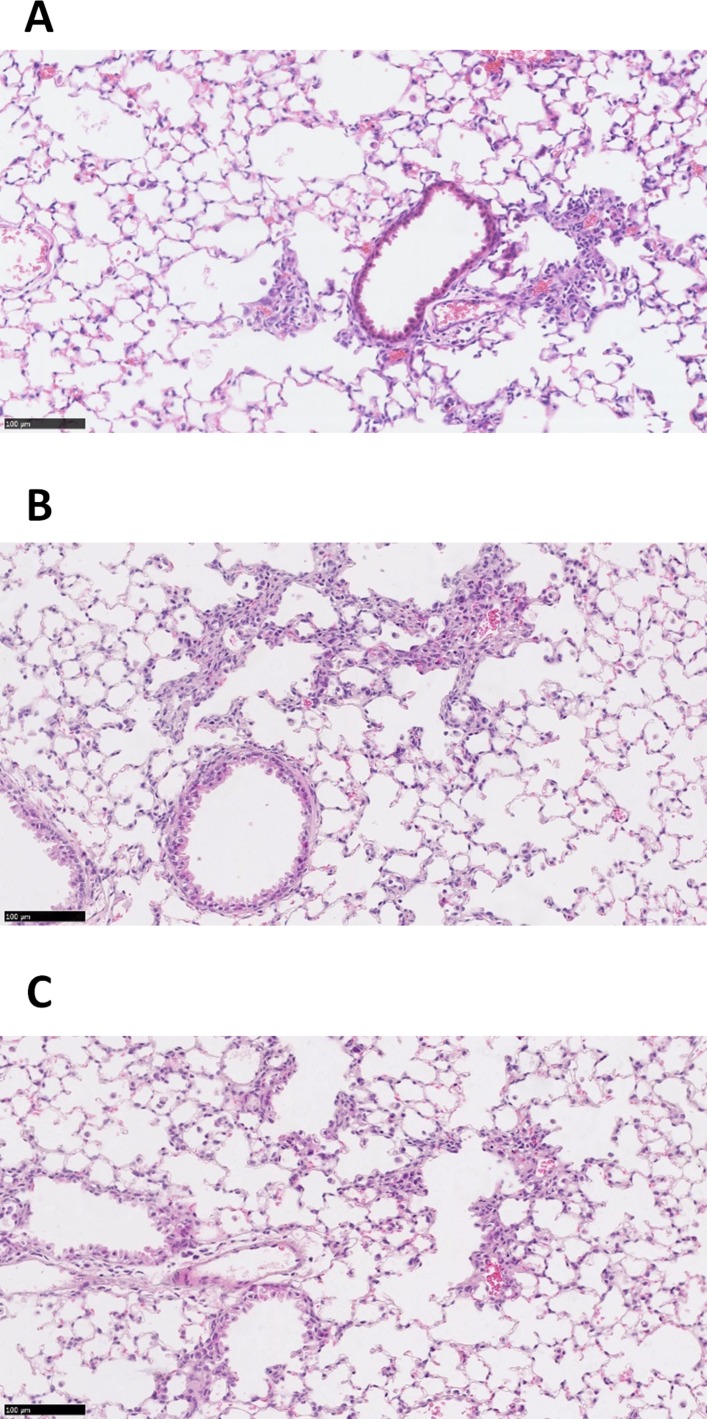
In vivo delivery of siRNA-containing nanocomplexes. Representative images of H&E stained murine lung sections following single (A) and triple (B) instillations of cationic αENaC siRNA nanocomplexes (n=3 for both), or a triple instillation (n=3) of cationic control siRNA nanocomplexes (C). All triple-instilled treated mice received three doses of nanocomplexes containing 16 µg siRNA on alternate days, and the lungs were harvested 48 hours after the third instillation. The mice that had one instillation of nanocomplexes containing 16 µg siRNA, also had their lungs harvested 48 hours following instillation. Scale bars=100 µm. siRNA, short interfering RNA.

The RTN siRNA formulations were well tolerated by the mice for single or triple dosing (online supplementary figure S6A,B). H&E staining of lung sections (n=3) showed that treatment with RTNs containing ENaC siRNA induced sporadic mild peribronchial cell infiltrates, the size and severity of which was unaffected by the number of instillations (figure 7A,B). Control siRNA induced a similar inflammatory response, although the foci were generally smaller and less frequent than with ENaC siRNA (figure 7C).

10.1136/thoraxjnl-2017-210670.supp6 Supplementary data

## Discussion

The lack of functional CFTR and hyperactivity of ENaC in the airways of patients with CF leads to disrupted ion and fluid homeostasis.^3^ Modulators of CFTR such as ivacaftor and the ivacaftor/lumacaftor (Orkambi) combination therapy offer treatment for patients with specific mutations,^34 35^ but there are some mutation classes where CFTR modulators will not be effective, such as nonsense mutations, and so other therapeutic strategies are required. The low-volume hypothesis of CF lung disease links increased activity of ENaC with ASL depletion in CF^3^ and so reducing transepithelial sodium absorption through ENaC is an alternative target for therapeutics.^11 36^ The role of ENaC in regulating ASL volume has been validated in recent work showing that inhibition of ENaC proteolytic activation using the SPLUNC1 or peptide fragments^37–39^ or QUB-TL1^40^ successfully prevented dehydration of the ASL in CF HBEC monolayers. Small molecule inhibitors of ENaC suffer from rapid systemic absorption from the lung and are associated with side effects such as hyperkalaemia due to ENaC inhibition in the kidney or pulmonary oedema.^11 41 42^ We have therefore investigated ENaC silencing mediated by nanoparticles carrying siRNA.

We used siRNA to target the major subunit, αENaC. The αENaC subunit forms a sodium-conducting pore, while the other two subunits enhance its activity.^43^ The α subunit is critical for sodium transport function and volume regulation as demonstrated in αENaC knockout mice, which die soon after birth due to a failure to clear their lungs of fluid.^44^ In addition, it has been shown that a low mRNA abundance of αENaC in the nasal epithelium of premature infants is linked with respiratory failure.^45^ Lastly, a mutation that leads to αENaC hyperactivity was found in patients with atypical CF.^46^

Nanoparticles offer protection to the siRNA during nebulisation and from nuclease attack in lung fluids and enable penetration of extracellular barriers such as mucus and the PCL^47^ before entering the epithelial cells by endocytosis. RTNs, comprising the oligolysine epithelial-targeting peptide (peptide E) and the liposome DOTMA/DOPE, self-assemble on mixing lipid and peptide components with siRNA to form cationic, monodisperse nanoparticles with a size of approximately 90 nm, which have been used previously for lung delivery of nucleic acids (including by nebulisation) in mice and pigs.^25 26 30 31 48^ Similar RTN formulations can package and transfect siRNA efficiently,^20 23 24 49^ and so we are now investigating use of αENaC siRNA to assess its therapeutic potential in epithelial lung models in vitro and in vivo.

We first assessed the ability of RTNs to penetrate mucus, which presents a physical barrier to nanoparticle siRNA delivery in the airways. Mucus is a gel-like layer, rich in charged mucin glycoproteins covering the lung epithelium and is particularly thick and sticky in the CF lung.^50 51^ Differentiated ALI cultures of human airway epithelial cells produce copious amounts of mucus, which is particularly viscous from CF cells.^52^ RTN diffusion in mucus from three sources was used including porcine gastric mucus and human mucus from differentiated CF and non-CF airway epithelial cultures. RTN diffusion rates were lowest in CF mucus, consistent with its increased viscosity. RTNs diffused at similar rates to siRNA alone in CF and non-CF mucus despite their size and charge differences. The diffusion rates of RTNs were greater than those of cationic PS nanoparticles of similar size used in cervicovaginal mucus,^53 54^ suggesting that surface properties of RTNs contribute to their mucus mobility.^32^

Silencing levels of αENaC by 30% from a single dose were achieved, which resulted in a significant reduction in amiloride-sensitive I_sc_ compared with control siRNA and untransfected cells. Silencing did not affect R_t_ indicating that there was no damage to the epithelial tight junctions by the nanoparticle complex directly or by decreased cell viability and that the changes in I_sc_ were a direct effect of reduced αENaC activity. Interestingly βENaC, but not γENaC, was also silenced by αENaC siRNA. This was observed in other studies,^17^ suggesting transcriptional feedback inhibition of βENaC by αENaC.

Enhancement of silencing of αENAC to 50% by three sequential transfections in CFBE cells reduced the transepithelial potential to normal range values (−7 mV) in ALI cultures.^55^ This may be a useful therapeutic biomarker as CF individuals display hyperpolarised nasal transepithelial potentials.^56^ Silencing of αENAC also caused a significant reduction in fluid absorption and increased ASL depth to normal levels. Increased hydration of the epithelial surface was also indicated by the reduced total protein concentration in mucus washings from αENaC siRNA-treated cultures for at least a week post-transfection, whereas the CFTR potentiator/corrector drug combination, VX-770 and VX-809, showed no effect on mucus protein concentration.

We further hypothesised that increased ASL depth would reduce impedance of ciliary motility. Indeed, we found that the CBF in ENaC siRNA-treated CF cells was increased to ~15 Hz, which compares well with normal CBF of approximately 16 Hz.^57^ Furthermore, the siRNA induced changes in CBF and fluid height after three doses, which was consistent with that reported for current drugs used to correct CFTR activity. VX-770 on its own increased CBF,^58^ whereas the VX-770 and VX-809 combination increased ASL height^59^ (both studies following five consecutive day treatments).

This study suggests that silencing ENaC in the range of 30%–50% is sufficient for restoration of epithelial ion transport balance, fluid transport and mucociliary properties. In previous αENaC silencing studies with siRNA, transfections were performed pre-ALI as transfections at ALI were ineffective,^18^ although they reported correction of the short circuit current and increased ASL when using siRNAs targeting both α and βENaC.^18^ Another study showed that silencing of αENaC with a commercial transfection reagent in submerged cultures lasted for at least 3 days, although they did not perform transfections at ALI.^13^ They also reported 35%–40% silencing in vivo with a liposomal formulation from a single administration that persisted at least 72 hours.^13^ Finally, an shRNA approach following lentiviral transduction of immortalised and primary cell lines used αENaC as their target.^12^ The authors transduced primary cells before the formation of tight junctions, unlike our fully differentiated cell approach, and showed up to 60% silencing. Similar to our findings, they have shown that reducing mRNA results in proportional changes in the short circuit current responses and also reduced net apical to basal fluid flux. In addition, they demonstrated that, at a high lentiviral dose, the βENaC mRNA was reduced, but γENaC was not, which is in agreement with our findings.

We finally quantified ENaC silencing, after single or repeat siRNA dosing (by oropharyngeal instillation) in the lungs of normal mice. The tolerance of repeated dosing supports the hypothesis that our nanoparticles do not induce neutralising antibodies, which is important for a potential life-long therapy. Minimal effective modulation of ENaC activity by repeated dosing of siRNA is likely to be much safer than complete inactivation of αENaC, since this could result in negative effects such as oedema observed in lungs of ENaC knockout mice.^44^ There were no significant adverse effects of repeated dosing of RTNs in mice as judged by body weight and behaviour. There was only minimal focal inflammation assessed histologically and the nanocomplex biodistribution was restricted to the lungs. Transfection experiments at ALI with human CFBE cells suggested that silencing of αENaC in the range of 30%–50% was sufficient for restoration of epithelial ion transport balance, fluid transport and mucociliary properties. We have shown that RTNs achieve these levels of silencing in mice and that the silencing is persistent for at least 1 week. These findings support the translational potential of this nanoparticle-mediated siRNA therapy for CF.

In summary, we have described nanoparticles that can penetrate mucus effectively and deliver siRNA to the airway epithelium and that silencing of αENaC can be achieved in vitro and in vivo leading to improved hydration and mucociliary function in CFBE monolayers. Other studies have also shown functional correction of airway epithelial cells but only by transfection prior to ALI culture. RTNs represent a powerful new tool for siRNA transfection studies in differentiated respiratory epithelial cells and in vivo. The potential to regulate ENaC to a minimally effective level, the persistence of silencing for at least 1 week and restriction of distribution to the lung after airway administration indicates translational potential and suggests advantages in efficacy and safety over orally administered, small molecule drugs.
