# Supplementary material for: Effective silencing of ENaC by siRNA delivered with epithelial-targeted nanocomplexes in human cystic fibrosis cells and in mouse lung
Source: Thorax. 2018 May 10;73(9):847–56. doi: 10.1136/thoraxjnl-2017-210670 (PMC6109249; doi:10.1136/thoraxjnl-2017-210670)
Supplement: Supplementary data [file thoraxjnl-2017-210670supp008.pdf]

Effective silencing of ENaC by siRNA delivered with epithelial-targeted nanocomplexes in human cystic fibrosis cells and in mouse lung

Aristides D. Tagalakis, Mustafa M. Munye, Rositsa Ivanova, Hanpeng Chen, Claire M. Smith, Ahmad M. Aldossary, Luca Z. Rosa, Dale Moulding, Josephine L. Barnes, Konstantinos N. Kafetzis, Stuart A. Jones, Deborah L. Baines, Guy W.J. Moss, Christopher O'Callaghan, Robin J. McAnulty, and Stephen L. Hart

ONLINE DATA SUPPLEMENT

Supplemental Methods

Materials

1,2-di-O-octadecenyl-3-trimethylammonium propane (DOTMA), 1,2-dipalmitoyl-*sn*-glycero-3-phosphoethanolamine-N-[methoxy(polyethylene glycol)-2000] (DPPE-PEG2000) and 1,2-dioleoyl-sn-glycero-3-phosphoethanolamine (DOPE) were purchased from Avanti Polar Lipids, Inc. (Alabaster, AL, USA). The targeting peptide E (K16GACSERSMNFCG) was synthesised by China Peptides (Shanghai, China). Pig stomachs were purchased from Mutch Meats (Whitney, UK). The normal mucus from a healthy subject and the human mucus from a CF patient collected through the MucilAir™ system were bought from Epithelix (Epithelix Sarl, Geneva, Switzerland). Amine modified polystyrene nanoparticles, sodium chloride, ethylene diamine tetra acetic acid (EDTA) and phenylmethyl sulfonyl fluoride (PMSF) were purchased from Sigma-Aldrich (Dorset, UK). The αENaC siRNA sequence (5'-GCAGUGAUGUUCCUGUUGA-3') was selected from an initial comparison of four candidate siRNAs in transfections of 16HBE14o- cells (as it gave the highest silencing) and were all obtained from Thermo Fisher Scientific (Northumberland, UK) for *in vitro* transfections. The αENaC siRNA for *in vivo* administration (gene id: Scnn1a - AM16830) was obtained from Life Technologies (Paisley, UK). Non-targeting control siRNA was obtained from Thermo Fisher Scientific (Northumberland, UK) and from Life Technologies (Paisley, UK). Cy-3 Silencer GAPDH siRNA was bought from Thermo Fisher Scientific (Northumberland, UK) and used for the mucus translocation studies. VX-770 and VX-809 were bought form Generon (Maidenhead, UK). GlyH-101 (1) was bought from Merck Chemicals (Nottingham, UK) and Forskolin from Cambridge Bioscience (Cambridge, UK). All other chemicals were bought from Sigma-Aldrich (Dorset, UK).

Liposome and Nanocomplex Formation

Lipids were dissolved in chloroform at 10 mg/mL then a lipid film was produced in a rotary evaporator by slowly evaporating the chloroform. Lipids were rehydrated with sterile, distilled water whilst constantly rotated overnight, and then sonicated in a water bath to reduce their size. Cationic liposomes made were: DOTMA:DOPE at 1:1 molar ratio.

Cationic receptor-targeted nanocomplex (RTN) formulations (at a weight ratio of 1:4:1, liposome: peptide: DNA or siRNA) were made by first adding the peptide to the liposome, followed by addition of the siRNA (or DNA) with rapid mixing and incubation for 30 minutes at room temperature (RT) to allow for complex formation. Non-targeted RTNs were made in the same way.

Particle Sizing and Zeta Potential Measurements

Nanocomplex preparations were diluted with distilled water to a final volume of 1 mL at a concentration of 5 μg/mL with respect to DNA or siRNA. They were then analysed for size and charge (ζ potential) using a Malvern Nano ZS (Malvern, UK). The following specifications were used: automatic sampling time of 10 measurements/sample, refractive index of 1.330, dielectric constant 78.5, viscosity 0.8872 cP and temperature of 25ºC. Dispersion Technology Software (DTS) version 5.03, which was provided by the manufacturer, was used for data processing.

Porcine Gastric Mucus Preparation

The stomachs of freshly slaughtered pigs were opened along their greater curvature, inverted, any food content removed mechanically. The stomachs were rinsed with double-distilled water

and the mucus lining was gently removed by scraping using a plastic spatula and transferred to a container containing a protease inhibiting buffer composed of 200 mM sodium chloride, 0.02 % (w/v) sodium azide, 5 mM EDTA and 1 mM PMSF. The mucus was mixed well with an equal volume of the protease inhibiting buffer and homogenised with a mixer for 2 min. The mucus-containing mixture was centrifuged at 11,200 *g* for 45 min at 4°C. The supernatant was poured into Visking dialysis tubing (MWCO: 12-14 kDa, Fisher Scientific, Loughborough, UK) and dialysed against deionised water for 24 h. The dialysed mucus solution was concentrated using an Amicon ultra-filtration stirred cell (Model 8400, 10 kDa membrane, Merck Millipore, UK) under nitrogen at a pressure of 40 psi and at 4°C. The retained mucus sample was collected into a bottle and frozen at -20°C until used.

Static Mucus Diffusion

Three different types of mucus were used: pig gastric mucus prepared in-house as above and non-CF and CF human mucus supplied commercially by Epithelix Sarl (Geneva, Switzerland). A 35 μm thick mucus barrier was established by application of 1 μl of pig gastric mucus, human CF or non-CF mucus to Transwell membranes (Costar, UK) and equilibrated at 37°C for 0.5 h. The mucus barrier thickness and confluence was confirmed using microscopy and the flux of sodium fluorescein demonstrated it was a rate limiting barrier. The translocation of the siRNA alone, the RTN and commercial cationic polystyrene nanoparticles was determined over time from an apical donor nanosuspension into an aqueous receiver fluid. 600 μL of Tris buffer pH 7.4 was used as the receiver fluid. 3 μL of nanoparticle suspensions containing Cy3-siRNA or siRNA alone at a concentration of 140 ng/μl were applied onto the surface of the barrier. At appropriate intervals, samples were removed from the receiver chamber and a ‘fit for purpose’

(in terms of precision and limit of detection) fluorescence spectroscopy method was used for detection at a maximum excitation of 550 nm and emission of 570 nm (FLx800 Microplate Fluorescence Reader, Bio-TEK Instruments, Swindon, UK). The cumulative concentrations (ng/cm^2) of translocated nanoparticles were quantified and plotted over time and the effective diffusion rates in mucus (D_m) using Fick's law

$$\frac{dM}{dt} = \frac{DC}{h}$$

where $\frac{dM}{dt}$ was the flux, D was the diffusion coefficient, C was the concentration of the permeant in the donor solution and h was the thickness of the barrier) and the effective diffusion rates in water (D_w) using Stokes' law

$$D = \frac{KT}{6\pi\eta r}$$

(where D is the diffusion coefficient, K is the Botlzman constant, T is the temperature, η is the viscosity and r is the particle radius) were also calculated.

Cell Culture and Transfections

The 16HBE14o- cells (kindly provided by Dieter Gruenert, California Pacific Medical Center Research Institute, San Francisco, CA, USA) were maintained in Minimum Essential Medium Eagle's modification (Sigma-Aldrich, Dorset, UK) at 37°C in a humidified atmosphere with 5% CO_2. Tissue culture medium was supplemented with 10% heat-inactivated foetal bovine serum (FBS, Life Technologies, Paisley, UK), 2 mM L-glutamine (Life Technologies) and 0.1 mM non-essential amino acids (Sigma-Aldrich, Dorset, UK). Cystic Fibrosis Bronchial Epithelial cells (primary CFBE) and Normal Human Bronchial Epithelial cells (primary NHBE) were

obtained from Epithelix (Epithelix SàRL, Genève, Switzerland). Primary cells were transduced in house (2) with a lentiviral vector expressing the anti-senescent *BMI-1* oncogene, obtaining CFBE-Bmi1 (CFBE) and NHBE-Bmi1 (NHBE) cells. Primary cells in submerged cultures were grown on 1% collagen-coated plastic flasks (PureCol® Bovine Collagen Solution, Type I, Advanced BioMatrix, San Diego, CA, USA) in Bronchial Epithelial Growth Medium (BEGM, Lonza) supplemented with Bovine Pituitary Extract, hydrocortisone, Human Epidermal Growth Factor, epinephrine, transferrin and insulin. Submerged cultures were transfected in 24 well plates (7x 10^4 cells per well) and transfected with nanocomplexes prepared as described above and diluted with OptiMEM (Life Technology, Paisley, UK) at different siRNA concentrations in replicates of 3. Plates were centrifuged at 400*g* for 5 min and then incubated for 4 h at 37°C. The transfection solution was then removed and replaced with complete media. The plate was then incubated for 48 h to determine expression of the gene.

For ALI culture, primary cells were grown in 12 mm collagen-coated transwell or snapwell inserts (Polyester (PET) Membrane Transwell-Clear Inserts, Corning, Corning Inc. Life Sciences, Tewksbury, MA, USA) at a seeding density of 1.5×10^5 or 1×10^6 viable cells per insert. Two days after seeding on transwells or snapwells, the BEGM media was removed from the apical side of the insert and the basolateral media changed to BEGM ALI medium (1:1 DMEM-Hi glucose: BEGM containing supplements) supplemented with 100 nM retinoic acid (Sigma-Aldrich, Dorset, UK). Medium was changed 3 times per week and mucus produced on the apical surface was removed once per week by gentle washing with PBS. Cells were transfected in snapwells with siRNA nanocomplexes at 100 nM final concentration (diluted with OptiMEM) by applying 1000 μl to the basolateral surface and 125 μl to the apical surface (no mucus was removed prior to transfection) and incubated for 4 hours at 37°C. The transfection

solution was then removed and replaced with ALI media. The plate was then incubated for 48 h or longer to determine gene expression or to perform functional studies. Transepithelial electrical resistance (R_t) of CFBE monolayers grown at ALI was measured at regular intervals using the EVOM Voltohmmeter (WPI, Hitchin, UK).

For certain transfections, 48h after the transfection or at different time points the apical surface was washed with 75 µl of PBS to collect the mucus. The amount of protein present in each mucus sample was determined with the Bio-Rad protein assay reagent (Bio-Rad Laboratories, Hemel Hempstead, UK) in a FLUOstar Optima luminometer (BMG Labtech, Aylesbury, UK).

Ussing Chamber Studies

Confluent CFBE monolayers were grown on snapwell clear membrane supports (Corning, UK) at ALI for 3 weeks and treated with or without nanoparticles. 48 h after treatment, they were then mounted into Ussing chambers and bathed from both sides with physiological saline containing (in mM) NaCl 117, $NaHCO_3$ 25, KCl 4.7, $MgSO_4$ 1.2, KH_2PO_4 1.2, $CaCl_2$ 2 and D-glucose 11 (pH 7.4). This was maintained at 37°C and gassed with a premixed gas (21% O_2 + 5% CO_2). Monolayers were maintained under open-circuit conditions and the spontaneous transepithelial potential (V_t) was monitored until a stable value was reached (~15 min) using a DVC 4000 voltage/current clamp and recorded via a PowerLab computer interface. Drugs were added in the order of amiloride (10 µM, apical), forskolin (25 µM, apical and basolateral) and GlyH-101 (10 µM, apical). NHBE cells were used as controls.

ASL Depth Measurement

CFBE cells grown in snapwells in ALI were transfected 3 times (every other day) with nanocomplexes containing siRNA to ENaC (n=4) or control siRNA (n=4) or were left untreated as controls (n=4). ASL depth (i.e. depth of both the mucus and PCL layers) was measured 72 h after the last transfection using confocal microscopy. The ASL in each snapwell was washed twice with PBS and then labeled with 20 μl PBS containing 2 mg/ml Rhodamine B isothiocyanate-dextran (10 kDa; Sigma-Aldrich, Dorset, UK) by apical application the day prior to the experiment. The following day the CFBE cells were stained using 5 μM Calcein-AM (Thermo Fisher Scientific, Northumberland, UK) dissolved in culture medium for at least 60 min and introduced to the basolateral compartment of the insert. Perfluorocarbon (300 μl; Sigma-Aldrich, Dorset, UK) was added to the apical compartment of the insert in order to prevent ASL evaporation. Fluorescent images of the epithelial layer and ASL height were obtained using a confocal microscope (Zeiss AxioObserver LSM 710 40x/NA1.2 plan-apochromat water objective, Jena, Germany). Samples were imaged in a #1.5 glass bottom dish (FD35, Fluorodish, World Precision Instruments, Hitchin, UK). Z stack acquisition used the optimal z-step as calculated in the Zen Software. For each snapwell, 5 different microscope fields randomly chosen were XZ scanned. Images were analysed with ImageJ/Fiji, using a macro to resliced images in an XZ orientation and threshold the ASL stain to produce a binary image for accurate manual height measurements. In each microscope field, the ASL height was measured in 9 separate regions randomly determined over the surface of the monolayer and then averaged.

Transepithelial Potential

Cystic fibrosis bronchial epithelial cells (CFBE), cultured in snapwells at ALI, were left untransfected or transfected 3 times (every other day) with nanocomplexes containing siRNA to

ENaC or control siRNA and then placed in an environmental chamber. The transepithelial potential (V_t) of these samples was measured with the pipette immersed in the ASL using a Scanning Ion Conductance Microscope (SICM; OpenIOLabs, Cambridge, UK) paired with an Axopatch 200B amplifier (Molecular Devices, CA, USA). V_t measurements were conducted using the amplifier in current clamp mode (I=0 mV). V_t was defined as the electrical potential between a high-resistance borosilicate pipette (R>80 MΩ) immersed directly into the ASL, and an Ag/AgCl bead electrode immersed in Ringer's solution on the basolateral side. In certain wells, the CFTR corrector VX-809 (10 µM) was added on the basolateral surface 1 day prior to ASL depth measurement, followed by addition of the potentiator VX-770 (10 µM) during measurement (again on the basolateral surface for approximately 20 min).

Transepithelial Fluid Transport Measurement

To quantify fluid absorption, CF cells were cultured in ALI as explained above. Following three sequential transfections (on alternate days) with ENaC or control siRNA, the apical surface of the cells was washed 48 h after the last transfection with 500 µl of a saline solution at RT containing (in mM): 137 NaCl, 2.7 KCl, 8.1 Na_2HPO4, 1.5 KH_2PO4, 1 $CaCl_2$, 0.5 $MgCl_2$. After washing, the apical side of the epithelium was covered with 150 µl of the same solution and 200 µl of mineral oil to prevent evaporation (3) and the cells returned to the incubator. After 24 h, the apical fluid was carefully removed, centrifuged to separate the mineral oil, and the volume of aqueous phase measured. The net flux across the epithelium is calculated as $J= (V_i\text{-}V_f)/A_t$, where V_i and V_f are the initial and final apical volumes, A is the = epithelium area, and t is the time interval between addition of V_i and recovering of remaining fluid V_f.

Assessment of Ciliary Beating

Respiratory primary CFBE cell cultures grown in snapwells in ALI were transfected 3 times (every other day) with nanocomplexes containing siRNA (αENaC or control) or treated with VX-770 and VX-809 as above or left untreated. The cultures were not washed for the duration of the experiment. 5 days after the last transfection they were placed in an incubation chamber (37°C, 5% CO_2) and were observed via an inverted microscope system (Nikon, UK) equipped with an ORCA-Flash4.0 V2 Digital CMOS camera (Hamamatsu, Japan). For each experimental condition, readings of ciliary beat frequency (CBF) were calculated from ten ciliated areas in the snapwell using ciliaFA software as previously described (4, 5).

Western Blot

Transfections for protein extraction were performed in a 6-well plate. The protein extraction procedure was performed on ice. Media was aspirated and cells were washed 3 times with ice-cold PBS. 30 µL of RIPA buffer supplemented with PMSF (Invitrogen, Paisley, UK) and protease inhibitor cocktail (Invitrogen, Paisley, UK) were added per well and incubated for 1 h on ice. Cells were then scraped and transferred in a 1.5 mL Eppendorf tube and kept on ice for 30 min vortexing every 10 minutes. Cells were then centrifuged at 14,000xg for 10 min at 4°C and the supernatants collected (cell lysates). The amount of protein in the lysates was measured with the bicinchoninic acid (BCA) protein assay (Thermo Fisher Scientific, Northumberland, UK) reading the absorbance at 595nm in a FLUOstar Optima luminometer (BMG Labtech, Aylesbury, UK).

Approximately 30 µg of the protein lysate were denatured at 95 °C for 5 min and loaded onto a 4-12% NuPAGE Bis-Tris pre-cast polyacrylamide gel. The gel was run at 100V for 15

min and 150V for 1 h in 1X NuPage running buffer. Proteins were transferred from the gel to a nitrocellulose membrane at 25V for 2.5 h in 1X NuPage transfer buffer (Life Technologies, Paisley, UK). The membrane was rinsed in di-ionised H_2O), washed 3x for 5 min in Tris-buffered saline/Tween (TBS-T) and subsequently incubated in a blocking buffer containing 5% milk powder in TBS-T for 1 h at RT. The membrane was then incubated with primary antibody in blocking buffer at 4 °C overnight. The primary antibodies used were polyclonal rabbit anti-α ENaC (PAI-920; Thermo Fisher Scientific, Northumberland, UK) at 1:750 dilution and mouse monoclonal anti-β-actin (AC-15; Sigma-Aldrich, Dorset, UK) at 1:5000 dilution. The membranes were then washed 3x for 5 min in TBS-T and incubated in secondary antibody in blocking buffer at RT for 1h. The secondary antibody for αENaC was polyclonal swine anti-rabbit HRP (horseradish peroxidase) at 1:1700 dilution (Dako, Ely, UK) and for β-actin polyclonal goat anti-mouse HRP at 1:1000 dilution (Dako, Ely, UK). Then the membranes were washed 3x for 5 min in TBS-T and developed with Clarity Western ECL substrate (1:1 of peroxidase and luminol/enhancer reagent; Bio-Rad, Hemel Hempstead, UK). The western blot densities were analysed using ImageJ software (NIH, Bethesda, USA).

In Vivo Delivery and Histology of Lung Tissues

Female C57Bl6 mice 6-8 week old were purchased from Charles River (Margate, UK). All procedures were approved by UCL animal care policies and were carried out under Home Office Licenses issued in accordance with the United Kingdom Animals (Scientific Procedures) Act 1986 (UK). Cationic nanocomplexes were prepared as described above at a final siRNA concentration of 0.29 mg/mL. Mice were instilled oropharyngeally following gaseous isoflurane induced anaesthesia (6, 7) with nanocomplexes in 55 µL (made in 5% glucose, v/v) containing

16 µg siRNA (αENaC or control siRNA), with untreated mice used as controls. In biodistribution studies, mice were instilled oropharyngeally as above with nanocomplexes in 55 µL (made in 5% glucose, v/v) containing 16 µg of siRNA-Dy677. Experiments were performed with replicates of 3 mice. 24 hours after injection, the mice were culled and organs (lung, liver, heart, kidneys, intestines and spleen) were resected and imaged using an IVIS Lumina Series III imaging system (PerkinElmer, Seer Green, UK). The images were processed using the Living Image software (PerkinElnmer, Seer Green, UK).

48 h or 7 days post-administration and the lungs excised and snap-frozen in liquid nitrogen and stored at -80°C till needed. In other studies siRNA-containing nanocomplexes were instilled 3 times (every other day) and the lungs excised 72 h after the 3rd instillation.

For the histology studies, mice were culled 48 h following oropharyngeal instillation of nanocomplexes and the lungs inflation fixed *in* situ with 4% paraformaldehyde at a pressure of 20 cm. The lungs were placed in 4% (w/v) PFA for 3 h followed by overnight incubation in 15% (w/v) sucrose/PBS and then a brief wash in 50% (v/v) ethanol and stored in 70% (v/v) prior to processing to paraffin wax.

RNA Extraction From Cells and Mouse Tissues

After 48 h, submerged cells were trypsinised and homogenised with Qiagen shredders (Qiagen, Crawley, UK). Cells grown on ALI were washed in PBS, scraped, centrifuged at 14,000*g* for 5 min and homogenised with Qiagen shredders. Total RNA was extracted from the homogenate using the RNeasy Kit (Qiagen, Crawley, UK), following the manufacturer's protocol and each sample underwent DNase treatment (Invitrogen, Paisley, UK) to eliminate any potential genomic DNA contamination. The samples were stored at -80 °C.

Mouse lungs were homogenised with the Precellys24 tissue homogenizer (2 cycles x 5600 rpm, 30 sec per cycle). Total RNA was extracted from mouse lysates using the RNeasy kit according to the manufacturer's instructions (Qiagen, Crawley, UK). RNA was checked for integrity using the Agilent 2100 Bioanalyzer (Wokingham, UK). All RNA samples had a RNA integrity number (RIN) of more than 9 indicating high quality RNA. Each sample underwent DNase treatment as above.

Quantitative Real-time PCR (qRT-PCR)

Total RNA (200 ng per reaction) was used in a one-step qRT-PCR (SensiFast Probe Hi-Rox One-Step Kit; Bioline, London, UK) that combines the reverse transcription with the quantitative PCR reaction. Human (Hs00168906_m1) or mouse (Mm01182998_g1) αENaC, human (Hs99999903_m1) or mouse β-actin (Mm00607939_s1), human βENaC (Hs01548617_m1) and human γENaC (Hs00168918_m1) were quantified by Taqman primers and probes (Thermo Fisher Scientific, Northumberland, UK). The qRT-PCR assays were performed in a Bio-Rad CFX96 Real-Time PCR Detection System with the following parameters: 45°C for 20 min, 95°C for 2 min and then 40 cycles at 95°C for 15 sec and 60°C for 1 min. Relative expression levels were calculated using the delta-delta Ct ($2^{-\Delta\Delta Ct}$) method (8).

Supplemental Figure Legends

Figure S1. Silencing efficiency of 16HBE14o- cells transfected with cationic ENaC siRNA nanocomplexes. **(A)** Representative western blot of αENaC protein from 16HBE14o- cells transfected with 75 nM of siRNA-containing nanocomplexes (n=3). αENaC major forms of 90 kDa and 65 kDa bands are shown in the blot. Untr= untreated cells. **(B)** The quantification by densitometry analysis of the % of remaining αENaC protein of the above blot is shown for the 65 kDa protein band of each group.

Figure S2. Expression of the different ENaC subunits in primary CFBE cells grown at ALI. CFBE cells were grown at ALI for 4 weeks and were then harvested and qRTPCR was performed in order to determine the expression of the different ENaC subunits. αENaC and βENaC are expressed relative to γENaC which is set at 1 (n=3). The middle horizontal lines represent the median values while the upper and lower horizontal bars represent the IQR. Asterisks indicate comparisons of specific formulations with statistical significance (***, $p<0.001$; Mann-Whitney non-parametric tests were performed).

Figure S3. Representative I_{sc} traces from untreated NHBE monolayers in Ussing chambers. This figure was used in work we conducted simultaneously and has been very recently published. (2)

Figure S4. Transepithelial electrical resistance (R_t) measurements on transfected and non-transfected CFBE-Bmi1 monolayers. The monolayers were cultured in snapwells and transfected with 100 nM of αENaC siRNA or control siRNA or were left untreated. 48 h later their R_t was

measured. The middle horizontal lines represent the median values while the upper and lower horizontal bars represent the IQR (n=3-7). There was no statistical difference between the groups tested (Mann-Whitney non-parametric tests were performed).

Figure S5. Radiant efficiencies (photons s^{-1} cm^{-2} $steradian^{-1}$ per μW cm^{-2}) of organs/tissues following oropharyngeal administration of nanocomplexes carrying Dy677-siRNA. 24 h later the mice were culled (n=3 per group) and organs (heart, lung, liver, kidneys, spleen and intestines) were extracted and imaged for fluorescence with the IVIS III system. The uptake of siRNA-Dy677 was significantly more in the lungs when compared to the intestines, the latter being the only other organ where fluorescence was detected. *, $p<0.05$ (Mann-Whitney non-parametric tests were performed to compare uptake differences between organs). Radiant efficiencies were measured using a Living Image 4.0 software package.

Figure S6. Weights of mice (in grams) **(A)** before and 7 days after a single instillation of nanocomplexes containing 16 µg siRNA and **(B)** during 3 instillations. Mann-Whitney non-parametric tests were performed. Each individual mouse is represented by a symbol. The lines are the means of the weights for each group of mice.

REFERENCES

1. Muanprasat C, Sonawane ND, Salinas D, Taddei A, Galietta LJ, Verkman AS. Discovery of glycine hydrazide pore-occluding CFTR inhibitors: mechanism, structure-activity analysis, and in vivo efficacy. *J Gen Physiol* 2004; 124: 125-137.
2. Munye MM, Shoemark A, Hirst RA, Delhove JM, Sharp TV, McKay TR, O'Callaghan C, Baines DL, Howe SJ, Hart SL. BMI-1 extends proliferative potential of human bronchial epithelial cells while retaining their mucociliary differentiation capacity. *Am J Physiol Lung Cell Mol Physiol* 2017; 312: L258-L267.
3. Gianotti A, Melani R, Caci E, Sondo E, Ravazzolo R, Galietta LJV, Zegarra-Moran O. Epithelial Sodium Channel Silencing as a Strategy to Correct the Airway Surface Fluid Deficit in Cystic Fibrosis. *Am J Resp Cell Mol* 2013; 49: 445-452.
4. Chilvers MA, O'Callaghan C. Analysis of ciliary beat pattern and beat frequency using digital high speed imaging: comparison with the photomultiplier and photodiode methods. *Thorax* 2000; 55: 314-317.
5. Smith CM, Djakow J, Free RC, Djakow P, Lonnen R, Williams G, Pohunek P, Hirst RA, Easton AJ, Andrew PW, O'Callaghan C. ciliaFA: a research tool for automated, high-throughput measurement of ciliary beat frequency using freely available software. *Cilia* 2012; 1: 14.
6. Du ZX, Munye MM, Tagalakis AD, Manunta MDI, Hart SL. The Role of the Helper Lipid on the DNA Transfection Efficiency of Lipopolyplex Formulations. *Sci Rep* 2014; 4: 7107.
7. Munye MM, Tagalakis AD, Barnes JL, Brown RE, McAnulty RJ, Howe SJ, Hart SL. Minicircle DNA Provides Enhanced and Prolonged Transgene Expression Following Airway Gene Transfer. *Sci Rep* 2016; 6: 23125.
8. Livak KJ, Schmittgen TD. Analysis of relative gene expression data using real-time quantitative PCR and the 2(T)(-Delta Delta C) method. *Methods* 2001; 25: 402-408.
