# Supplementary material for: Effective silencing of ENaC by siRNA delivered with epithelial-targeted nanocomplexes in human cystic fibrosis cells and in mouse lung
Source: Thorax. 2018 May 10;73(9):847–56. doi: 10.1136/thoraxjnl-2017-210670 (PMC6109249; doi:10.1136/thoraxjnl-2017-210670)
Supplement: Supplementary data [file thoraxjnl-2017-210670supp007.pdf]

Formulation	Normal human mucus (ng/cm^2)	Cystic Fibrosis mucus (ng/cm^2)	Pig Gastric mucus (ng/cm^2)
**RTN**	961.5 ± 44.8	441.1 ± 52.7	1046.1 ± 47.6
**siRNA**	917.3 ± 60.1	390.1 ± 15.8	472.0 ± 8.8
**Polystyrene beads**	not done	100.0 ± 57.6	782.7 ± 123.0

Table S1. Nanoparticle translocation through mucus. Cumulative concentrations through different mucus sources of the receptor-targeted nanocomplexes (RTN), siRNA and polystyrene nanoparticles (PS) were determined from the accumulated fluorescence over a period of 1 hour. Values are averages ± SEM.

αENaC siRNA dose	αENaC silencing %	βENaC silencing %	γENaC silencing %
**Single**	30	51	0
**Triple**	54	not done	not done

Table S2. ***In vitro*** **siRNA silencing of ENaC in epithelial cells.** CFBE cells grown at ALI were transfected with RTN formulations containing either αENaC siRNA or control siRNA at 100 nM. The percentage of silencing of α, β and γ ENaC subunits was calculated 48 h after transfection (n=3 per formulation). Values shown are the differences of the median silencing of ENaC siRNA treated group compared to their respective control siRNA group.

αENaC siRNA dose	αENaC	Control siRNA	Untreated
Amiloride-sensitive (I_{sc}) μA/cm^2	6.4 (IQR: 5.4-9.8)	11.5 (IQR: 10.1-14.1)	14.3 (IQR: 13.2-17.9)
R_t Ω cm^2	693.5 (IQR: 565.7-805.8)	783.2 (IQR: 711.1-953.2)	676.7 (IQR: 653.1-744.2)

Table S3. The effects of siRNA transfection on amiloride-sensitive short circuit current I_{sc} and transepithelial electrical resistance (R_t). Values are medians with the Interquartile range (IQR).

Analysis	αENaC siRNA	Control siRNA	VX-770/ VX-809 CFBE	NHBE untreated	CFBE untreated
**Transepithelial potential (V_t), mV**	-7.2 (IQR: -5.7 to -12.2)	-16.0 (IQR: -14.8 to -19.0)	-6.8 (IQR: -6.7 to -12.7)	-7.7 (IQR: -6.3 to -11.6)	not done
**ASL depth, µm**	12.1 (IQR: 10.7-14.9)	7.9 (IQR: 6.4-9.8)	not done	not done	8.2 (IQR: 5.8-11.1)
**Ciliary beat frequency, Hz**	14.5 ± 0.5	11.9 ± 1.0	12.8 ± 0.5	not done	9.6 ± 0.7
**Net fluid absorption, µl/cm^2/h**	0.9 (IQR: 0.6-1.3)	1.6 (IQR: 1.3-1.9)	not done	not done	1.5 (IQR: 1.1-1.9)

Table S4. Effects of triple-dose αENaC silencing on transepithelial potential (V_t), airway surface liquid depth (ASL), ciliary beat frequency and net fluid absorption. Values are shown as mean ± SEM for ciliary beat frequencies. All other values are medians with the Interquartile range (IQR).

siRNA dose	% αENaC silencing 48h-72h	% αENaC silencing, 1 week after administration
**Single**	30	23
**Triple**	58	not done

Table S5. *In vivo* silencing of αENaC. Following αENaC siRNA administration, silencing was determined by qRT-PCR at 48 h and 1 week. Triple dosing was also performed and the percentage of silencing was calculated 72 after the final dose. Values shown are the differences of the median silencing of αENaC siRNA treated group compared to their respective control siRNA group.
