# Supplementary figures and images for: Effective silencing of ENaC by siRNA delivered with epithelial-targeted nanocomplexes in human cystic fibrosis cells and in mouse lung
Source: Thorax. 2018 May 10;73(9):847–56. doi: 10.1136/thoraxjnl-2017-210670 (PMC6109249; doi:10.1136/thoraxjnl-2017-210670)

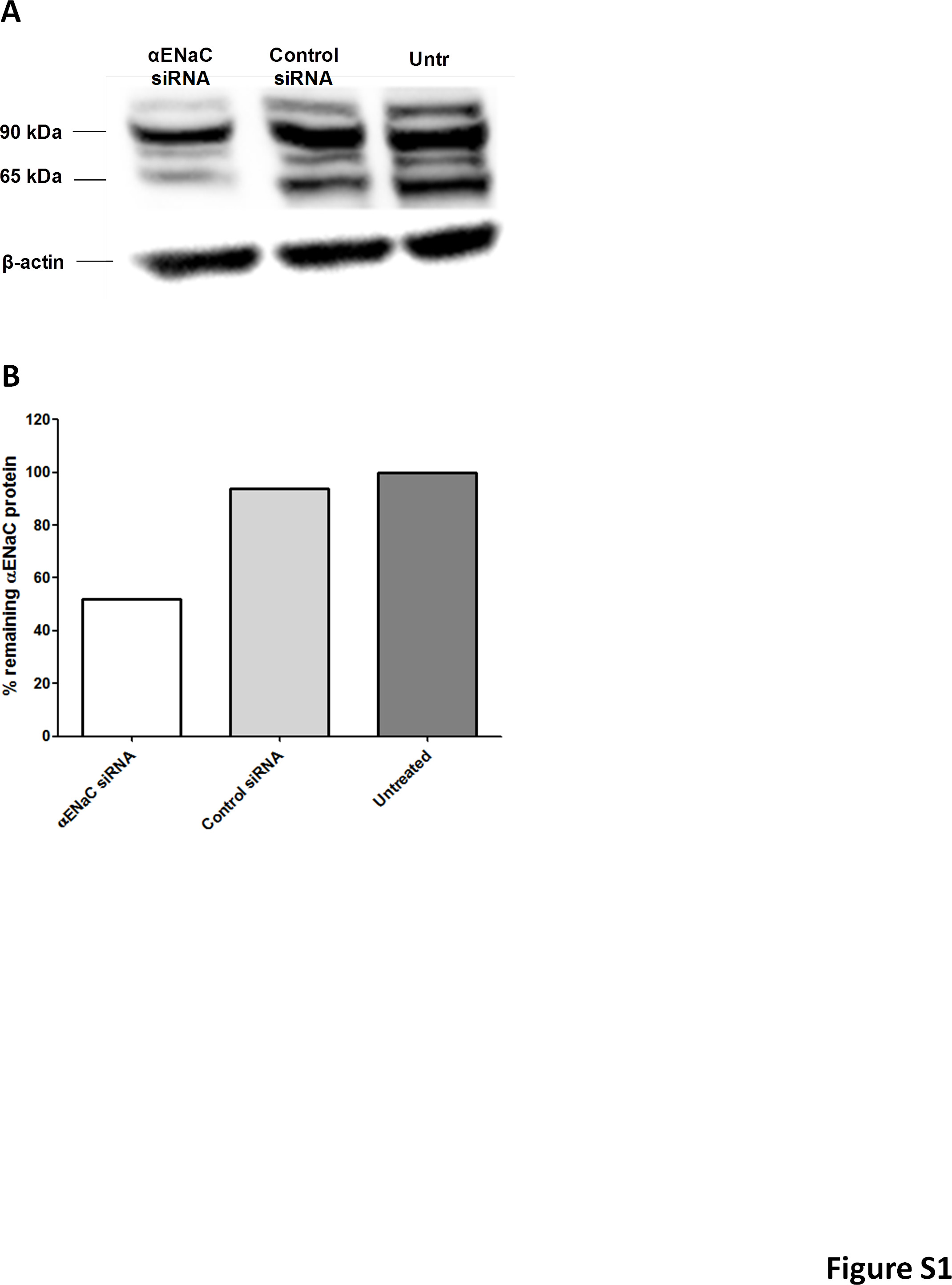

Supplement: Supplementary data [file thoraxjnl-2017-210670supp001.jpg]

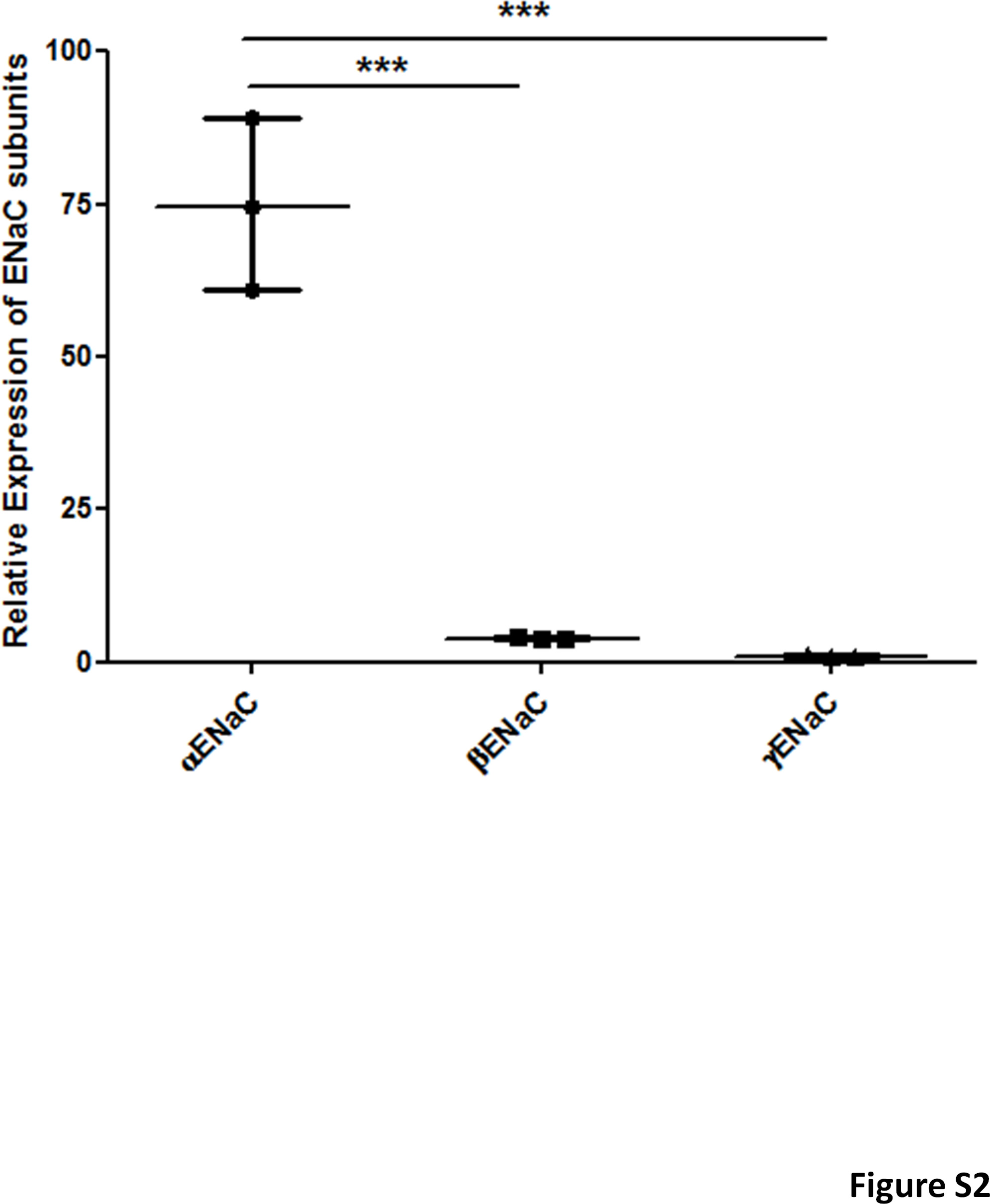

Supplement: Supplementary data [file thoraxjnl-2017-210670supp002.jpg]

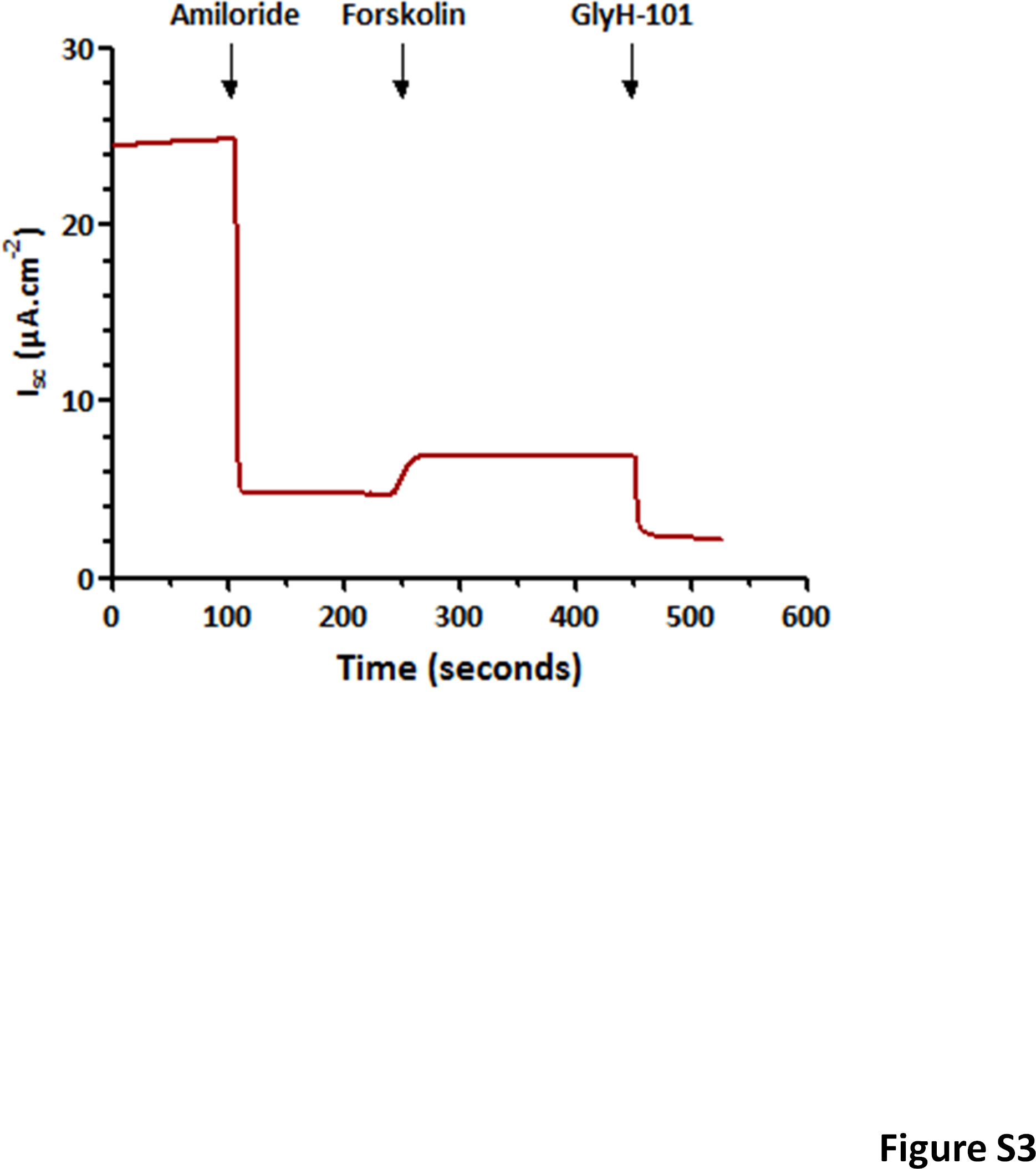

Supplement: Supplementary data [file thoraxjnl-2017-210670supp003.jpg]

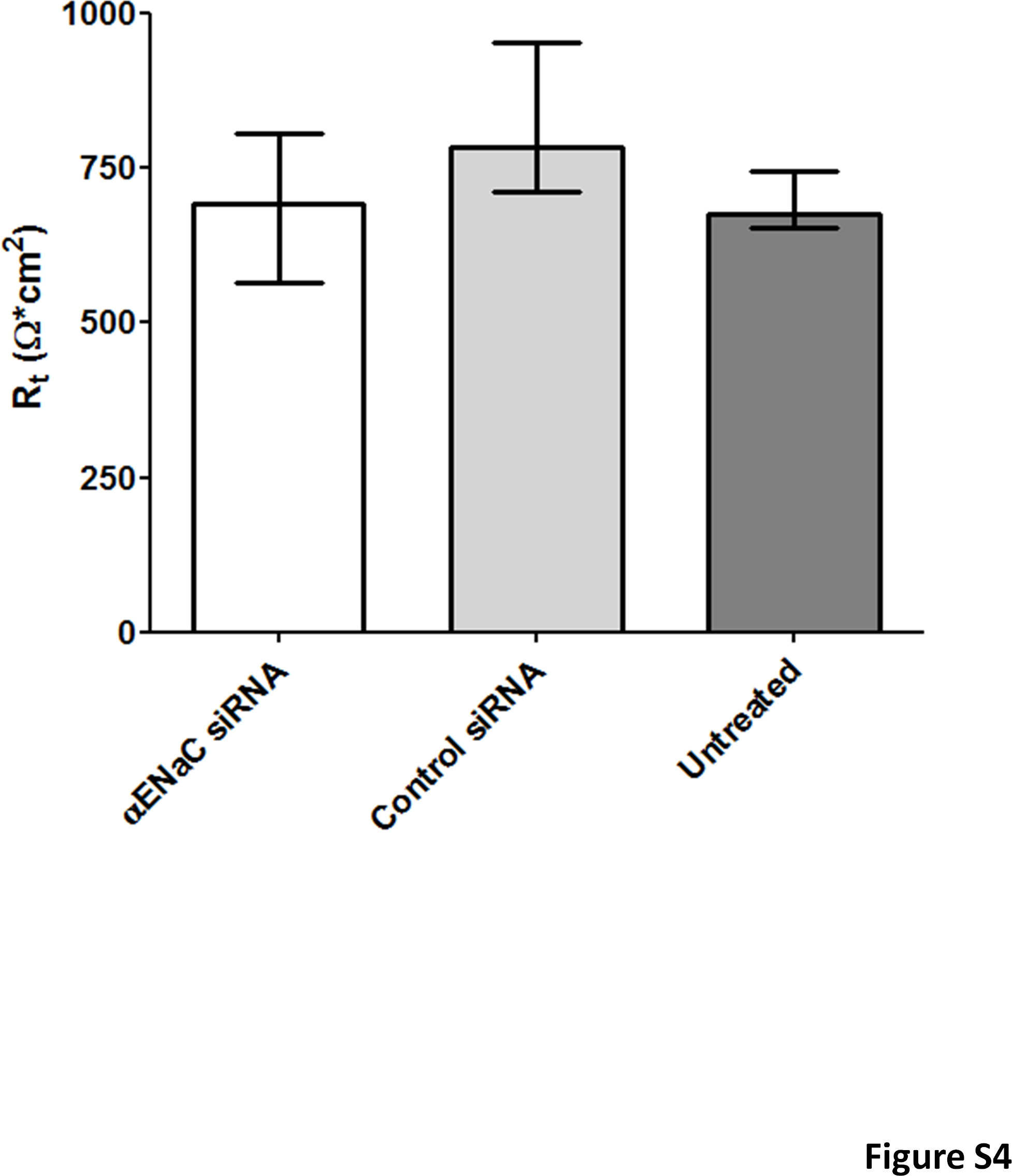

Supplement: Supplementary data [file thoraxjnl-2017-210670supp004.jpg]

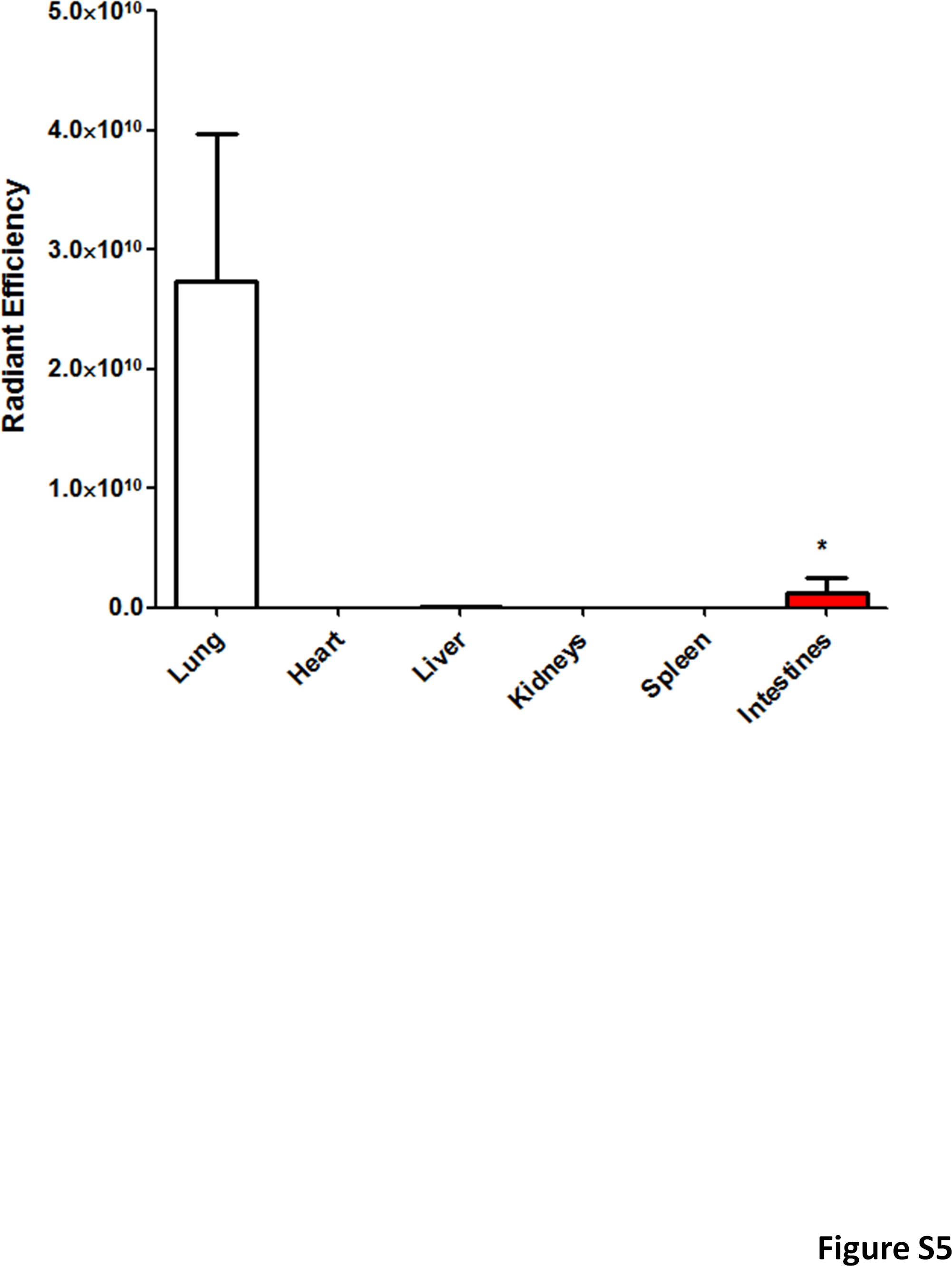

Supplement: Supplementary data [file thoraxjnl-2017-210670supp005.jpg]

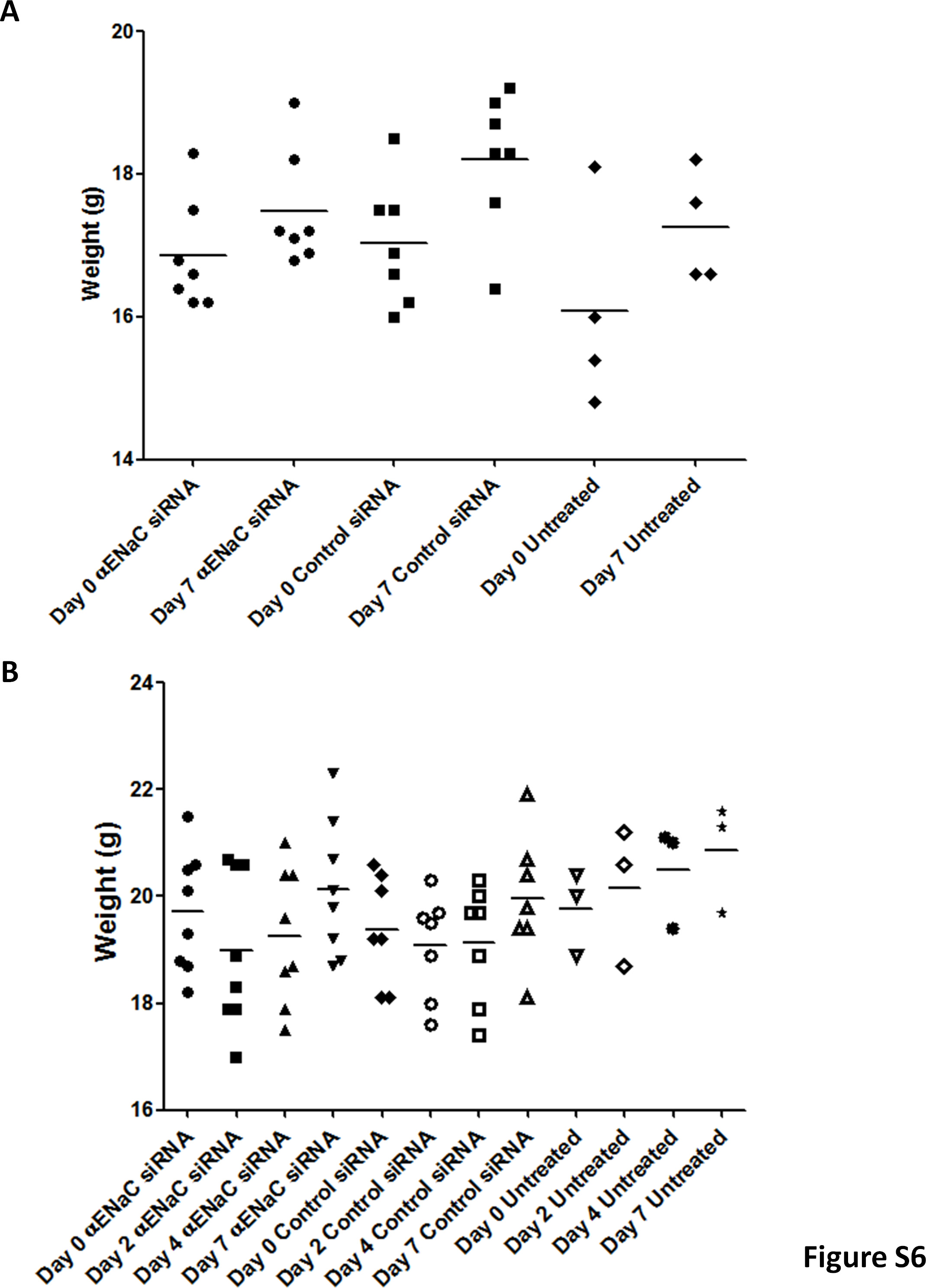

Supplement: Supplementary data [file thoraxjnl-2017-210670supp006.jpg]
